# Coverage Analysis of Hybrid FSO/RF Communication Systems with Selection Combining in High-Speed Railway Scenarios

**DOI:** 10.3390/s26165149

**Published:** 2026-08-14

**Authors:** Xin Zhang, Shuai Dong, Xin-Run Yan, Zheng Li, Yuan-Yuan Li, Jin-Yuan Wang

**Affiliations:** 1 School of Communications and Information Engineering, Nanjing University of Posts and Telecommunications, Nanjing 210003, China; 1224014641@njupt.edu.cn (X.Z.); 1223014125@njupt.edu.cn (S.D.); 1024010404@njupt.edu.cn (X.-R.Y.); 1024010221@njupt.edu.cn (Z.L.); 1024010220@njupt.edu.cn (Y.-Y.L.); 2 Key Laboratory of Opto-Technology and Intelligent Control, Ministry of Education, Lanzhou Jiaotong University, Lanzhou 730070, China

**Keywords:** high-speed railway communications, hybrid FSO/RF system, selection combining, coverage analysis, edge coverage probability, percentage of cell coverage area

## Abstract

Modern transportation systems impose stringent requirements on high-speed railway (HSR) communications in terms of high data rate and high reliability. Conventional radio frequency (RF) links are limited by the scarcity of spectrum resources, whereas free-space optical (FSO) links are highly vulnerable to atmospheric turbulence and weather conditions. To overcome the limitations of a single transmission medium, this paper proposes and investigates a hybrid FSO/RF selection combining (SC) communication system for narrow-strip cells in HSR scenarios. First, a geometric coverage model is established for heterogeneous base-station deployment, together with a location-aware activation mechanism that enables adaptive activation of base stations according to train positions. Then, by taking practical railway operating conditions into account, the FSO link is modeled to capture the combined effects of atmospheric turbulence, pointing errors, and atmospheric attenuation, while the RF link is modeled as a composite channel incorporating path loss, shadow fading and small-scale fading. Based on this framework, exact closed-form expressions for the system edge coverage probability (ECP) and the percentage of cell coverage area (CCA) are derived. For the sake of comparisons, theoretical expressions of the standalone FSO and standalone RF links are also obtained. Finally, Monte-Carlo simulations are conducted to verify the validity of the derived analytical results. The numerical comparisons demonstrate excellent agreement between the analytical predictions and simulation results, with average relative deviations of only 0.047% for ECP and 0.095% for the percentage of CCA. Compared with either the standalone FSO system or standalone RF system, the proposed hybrid architecture achieves superior coverage performance across different transmit powers, weather conditions, and cell diameters, offering a highly reliable and robust networking solution for future seamless broadband HSR communication systems.

## 1. Introduction

With the rapid advancement of urbanization and the growing demand for convenient transportation, high-speed railway (HSR) has become an indispensable component of modern transportation networks. Meanwhile, the continuous development of intelligent railway services and the explosive growth of passenger data traffic have imposed increasingly stringent requirements on the transmission capacity, reliability, and quality of service of railway communication systems. As the successor to global system for mobile communications–railway, the future railway mobile communication system, which is built upon fifth-generation (5G) technologies, aims to provide high-data-rate, low-latency, and highly reliable communication services for intelligent railway applications. However, current railway broadband access systems still primarily rely on radio frequency (RF) communications to provide network connectivity for passengers [1]. With the continuous increase in train operating speeds and communication traffic demands, conventional RF communications suffer from limited spectrum resources and complex electromagnetic environments [2], resulting in significant challenges in terms of transmission capacity and coverage performance. Consequently, exploring optical wireless communication technologies capable of providing ultra-large bandwidth, high-speed transmission, and strong immunity to electromagnetic interference has emerged as an important research direction for future intelligent railway communication systems.

In recent years, free-space optical (FSO) communication has attracted extensive attention from both academia and industry as a highly promising technology for broadband wireless access. Owing to its advantages of high bandwidth [3,4], license-free operation [5], and strong anti-interference capability, FSO has gradually emerged as an ideal candidate for addressing communication challenges in HSR systems and has shown great potential for achieving high-speed communication in high-mobility railway environments [6]. However, the line-of-sight transmission nature of FSO links makes them highly sensitive to atmospheric conditions. Atmospheric attenuation caused by fog and haze, as well as atmospheric turbulence induced by aerosol scattering, can lead to beam spreading, beam wandering, and intensity fluctuations, severely degrading the reliability of FSO communications [7,8]. In addition, in HSR scenarios, the high mobility of the train and track irregularities inevitably introduce pointing errors between the transmitter and receiver, which further deteriorate the transmission performance of the FSO link.

To overcome the reliability bottleneck of standalone FSO systems under adverse weather conditions or misalignment errors, combining FSO with conventional RF systems to form a hybrid FSO/RF communication system has become a highly attractive and efficient solution. At present, research on hybrid FSO/RF systems has covered a wide range of frontier topics. In the area of physical layer security, researchers have analyzed secrecy performance under various fading channels [9,10]. In terms of system architecture and coding design, space modulation, multiple-input multiple-output, and rate-compatible low-density parity-check codes have been introduced to improve transmission efficiency [11,12]. Moreover, hybrid FSO/RF techniques have also been extensively investigated in complex scenarios such as two-hop cooperative systems, wireless-powered relays, and air-to-ground integrated networks [13,14,15,16]. To optimize reception performance, a variety of diversity combining schemes, such as maximal-ratio combining, adaptive combining, and selection combining (SC), have been thoroughly evaluated and compared [17,18,19,20]. In heterogeneous FSO/RF systems, SC is considered a practical receiver strategy because it avoids direct signal-level fusion between optical and RF receivers and only requires link-quality-based selection. However, most existing studies on hybrid FSO/RF systems are still limited to fixed point-to-point communications, static networks, or high-altitude platforms, and few studies have introduced such a promising hybrid architecture into HSR scenarios characterized by extremely high mobility and special geometric topology.

In wireless communication system design, cell coverage analysis is a core task for evaluating service quality and guiding base-station deployment. The key metrics used to assess coverage performance mainly include the edge coverage probability (ECP) and the percentage of cell coverage area (CCA). Unlike the circular cells in conventional cellular networks, HSR systems are constrained by the linear topology of railways and therefore require a narrow-strip cell coverage model. At present, some progress has been made in the analysis of coverage performance for narrow-strip cells in HSR scenarios. For example, researchers have extensively analyzed the narrowband coverage performance of conventional RF networks under the Suzuki composite fading channel, which includes path loss, log-normal shadowing, and Rayleigh small-scale fading [21], as well as the coverage optimization problem in HSR systems employing carrier aggregation techniques [22]. Similarly, coverage analysis of single-FSO HSR cells has also begun to attract attention [6]. However, for hybrid FSO/RF systems applied to narrow-strip HSR cells, coverage performance analysis, especially the derivation of ECP and the percentage of CCA, remains largely unexplored in the literature. Considering the deterministic railway trajectory and the complementary propagation characteristics of FSO and RF links, how to deploy heterogeneous base stations and dynamically activate them according to train locations to effectively exploit link diversity for continuous coverage remains a challenge of both theoretical significance and practical engineering value.

To fill this research gap, this paper introduces a hybrid FSO/RF communication architecture into intelligent HSR scenarios. We design a heterogeneous network deployment scheme suitable for narrow-strip HSR cells, in which FSO base stations are deployed relatively densely while RF base stations provide wider single-point coverage. To guarantee communication continuity while offering potential energy-saving benefits, the proposed system adopts parallel transmission and a location-aware activation strategy, where data streams from the optical backbone are simultaneously transmitted over different physical links and non-serving base stations remain in sleep mode. Considering that the onboard front-end link is similar to conventional cellular communications, the focus of this paper is strictly confined to the complex high-mobility train-to-ground backhaul link. At the train receiver, an SC strategy is employed to dynamically select the link with better instantaneous signal quality. Based on this architecture, this paper conducts rigorous mathematical modeling and theoretical analysis of the coverage performance of the hybrid FSO/RF backhaul link in narrow-strip cells. The main contributions of this paper are summarized as follows:

- For high-speed railway narrow-strip cell scenarios, a hybrid FSO/RF communication architecture is proposed. The system adopts heterogeneous deployment of FSO and RF base stations with a shared optical fiber backbone, together with a location-aware dynamic activation mechanism, which improves coverage reliability while providing potential energy-saving benefits. In addition, a relatively complete hybrid FSO/RF channel model is developed, providing a tractable mathematical foundation for analytical performance evaluation and enhancing the ability to characterize the complex propagation environment in high-speed railway scenarios.
- A closed-form ECP expression for the hybrid FSO/RF system is derived by characterizing the statistical behavior of the received signal-to-noise ratio (SNR) and utilizing Meijer G-function representations, which enables efficient analytical evaluation of the cell-edge coverage performance. Meanwhile, closed-form expressions for single FSO and single RF links are also obtained, which serve as analytical benchmarks for quantifying the coverage gain of the proposed hybrid scheme.
- To address the intractability of evaluating the percentage of CCA, a compact power-law approximation method is proposed, which overcomes the analytical difficulty caused by high-order nonlinear integrals and yields a highly accurate closed-form expression for the percentage of CCA. The derived analytical results are validated by Monte Carlo simulations, demonstrating the effectiveness of the proposed coverage analysis framework. The proposed hybrid architecture demonstrates significant performance gains over standalone FSO and RF systems in terms of both ECP and the percentage of CCA. Moreover, by avoiding unnecessary activation of non-serving base stations, the proposed location-aware activation mechanism provides potential benefits in reducing operational overhead, thereby offering useful design insights for coverage planning and efficient deployment of HSR communication networks.

The paper is structured as follows. Section 2 describes the proposed system architecture and channel models. The derivation of the ECP and the percentage of CCA expressions is provided in Section 3. Section 4 presents numerical evaluations and related discussions. Finally, Section 5 summarizes the main findings of this work.

## 2. System and Channel Models

### 2.1. System Model

**Figure 1:**
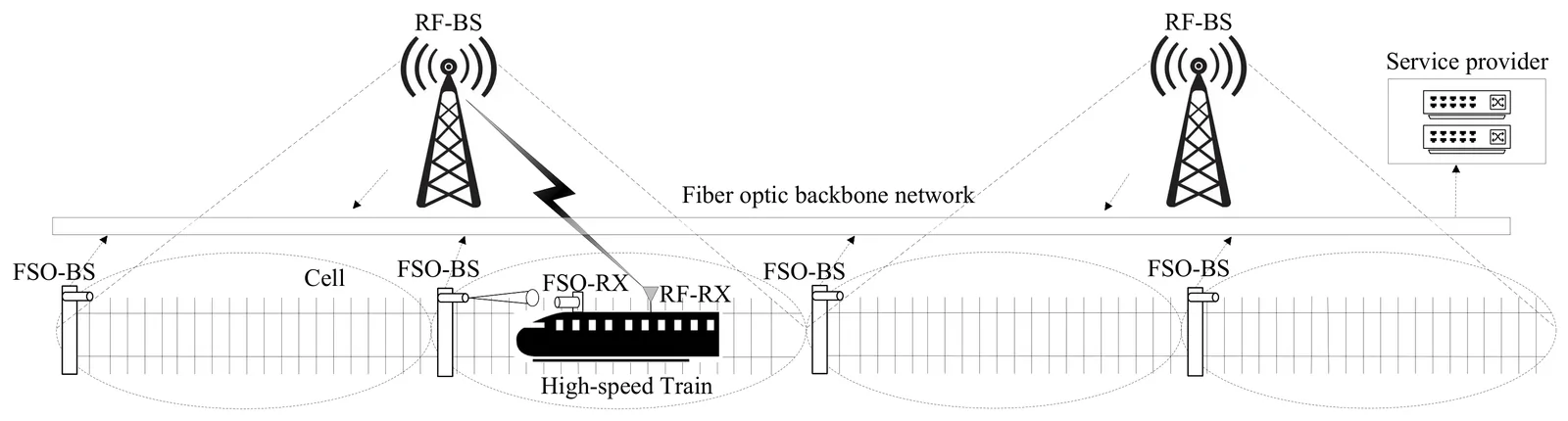
A hybrid FSO/RF communication system for narrow-strip HSR cells.

As shown in Figure 1, this paper considers a hybrid FSO/RF communication system applied to narrow-strip cells in HSR scenarios. In the proposed architecture, FSO base stations (FSO-BSs) and RF base stations (RF-BSs) are heterogeneously deployed along the railway line and share the same high-capacity optical backbone. Specifically, the FSO-BSs are deployed relatively densely, with the spacing between adjacent base stations set to the cell diameter. In contrast, owing to the use of directional antennas pointing to both sides of the track, each RF-BS provides a wider single-point coverage area, and thus the deployment spacing is set to twice the cell diameter. Such a heterogeneous network architecture enables the entire HSR line to be fully and continuously covered by a sequence of narrow-strip cells.

In addition to the communication infrastructure, sensing devices are considered in the proposed system to provide real-time train position and environmental information. Specifically, onboard positioning sensors provide the train location information, while trackside visibility sensors measure the atmospheric visibility distance. These sensing data provide the necessary geometric and environmental parameters for establishing the proposed hybrid FSO/RF communication system model.

To ensure communication continuity and high reliability under high-mobility train operation, the system adopts parallel transmission and a location-aware activation mechanism. Specifically, the train position information obtained from onboard positioning sensors is utilized to determine the activation state of along-track base stations. The data streams from the service provider are first delivered through the optical backbone to both the FSO and RF base stations, which then transmit the same signal streams to the train over different physical links at the same data rate. Specifically, the along-track FSO/RF base stations are interconnected through the shared optical backbone network, which connects heterogeneous access nodes with a centralized network controller and carries control signaling. The train periodically reports its position information obtained from onboard positioning sensors to the centralized network controller. Based on the train position information, railway trajectory, and predefined deployment locations of base stations, the controller determines the upcoming service region and sends activation commands to the corresponding FSO/RF base stations before the train enters their coverage areas. Meanwhile, base stations outside the current service region remain in sleep mode to avoid unnecessary continuous operation. High-accuracy train position information is assumed based on onboard positioning sensors, while positioning errors in practical systems may exist and affect the accuracy of BS activation decisions. This location-aware activation mechanism enables coordinated management of heterogeneous access nodes and provides potential benefits in reducing operational overhead by avoiding continuous activation of non-serving base stations.

On the train side, rooftop-mounted receivers are installed for both the FSO and RF links: an FSO receiver (FSO-RX) and an RF receiver (RF-RX). After capturing the spatial signals, the receiver employs an SC strategy to continuously evaluate the instantaneous signal quality of the two links and dynamically select the one with better performance for data reception. It is worth noting that the adoption of SC is motivated by practical implementation considerations in heterogeneous FSO/RF HSR systems. Since FSO and RF links employ different transmission mechanisms and receiver structures, SC does not require direct signal-level fusion between heterogeneous links and provides a low-complexity reception strategy suitable for high-mobility HSR scenarios. Moreover, the use of SC at the receiver eliminates the need for strict phase synchronization between the two heterogeneous links. The distributed FSO/RF base stations are assumed to achieve time synchronization through standard synchronization protocols (e.g., IEEE 1588 Precision Time Protocol) over the shared optical backbone network, where packet/frame-level synchronization is sufficient for coordinated operation of heterogeneous access nodes. The demodulated data are then forwarded to the in-train communication network.

The overall HSR communication link can be divided into two main parts: the train-to-ground backhaul link, i.e., the link from the base station to the train receiver, and the in-train front-end link, i.e., the link from the onboard access point to the user terminals. Since the communication mechanism of the in-train front-end link is similar to that of conventional cellular or indoor Wi-Fi systems that have been extensively studied, the main technical bottleneck in HSR communications lies in the train-to-ground backhaul link under high-mobility conditions. Therefore, the focus of this paper is strictly confined to the performance analysis of the backhaul link. For uplink transmission, another type of communication (such as WiFi, Li-Fi, 5G, etc.) can be used to avoid interference.

**Figure 2:**
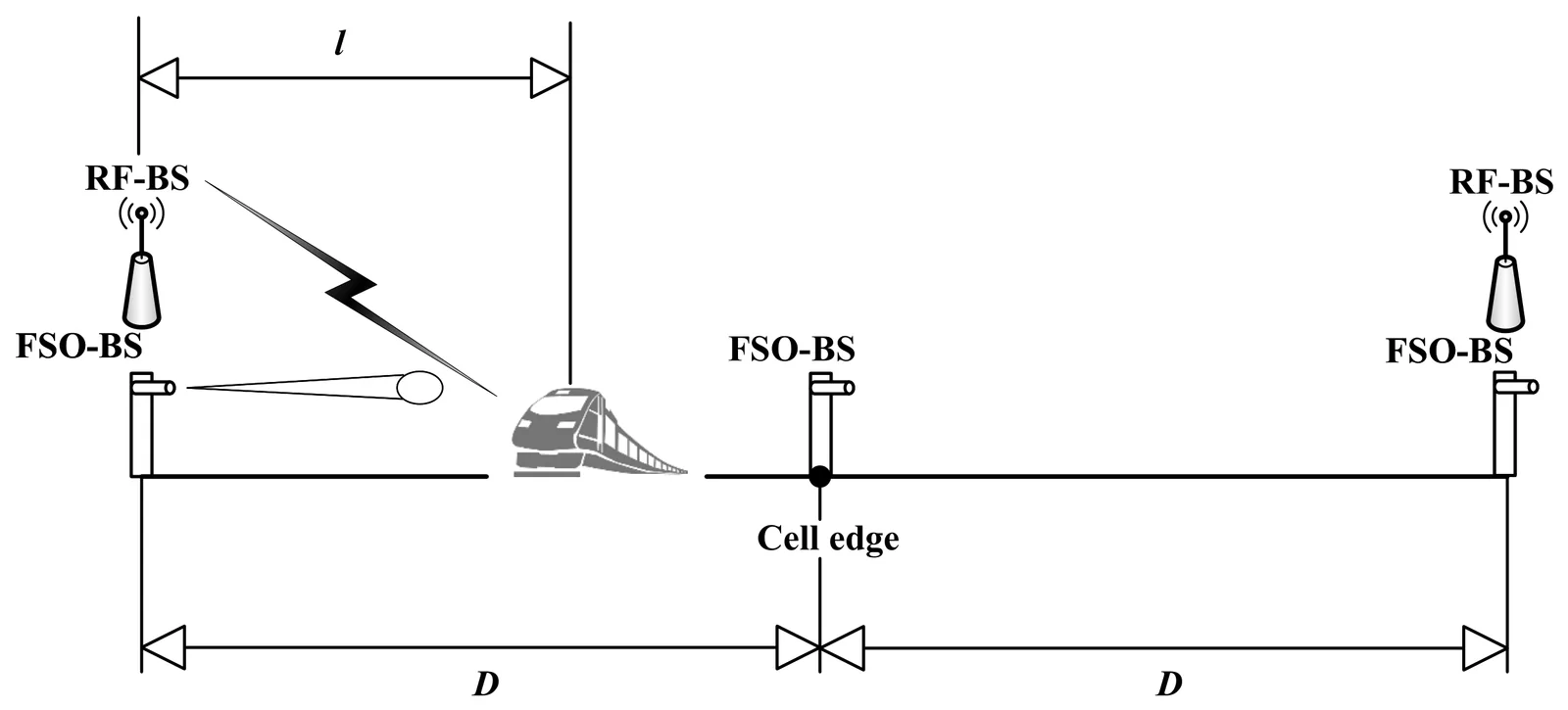
Cell coverage scenario.

Figure 2 illustrates the cell coverage and geometric distance model of the proposed hybrid system. Since both the FSO-BSs and RF-BSs are deployed in close proximity to the railway track, the perpendicular distance between the base stations and the track is extremely small and can be neglected. In addition, although the FSO transmitter is installed slightly higher than the roof-mounted receiver on the train, the propagation path of the laser beam can be approximated as horizontal line-of-sight transmission over sufficiently long communication distances. Under these assumptions, the three-dimensional transmission distance can be reasonably simplified to a one-dimensional distance. This approximation captures the dominant longitudinal propagation characteristics of HSR communications. As shown in Figure 2, the cell diameter, which is also equal to the spacing between two adjacent FSO-BSs, is denoted by *D*, and the instantaneous horizontal distance between the train receiver and the currently active serving base station is denoted by *l*, which can be determined based on the train position information obtained from the onboard positioning sensors.

### 2.2. FSO Subsystem

**Figure 3:**
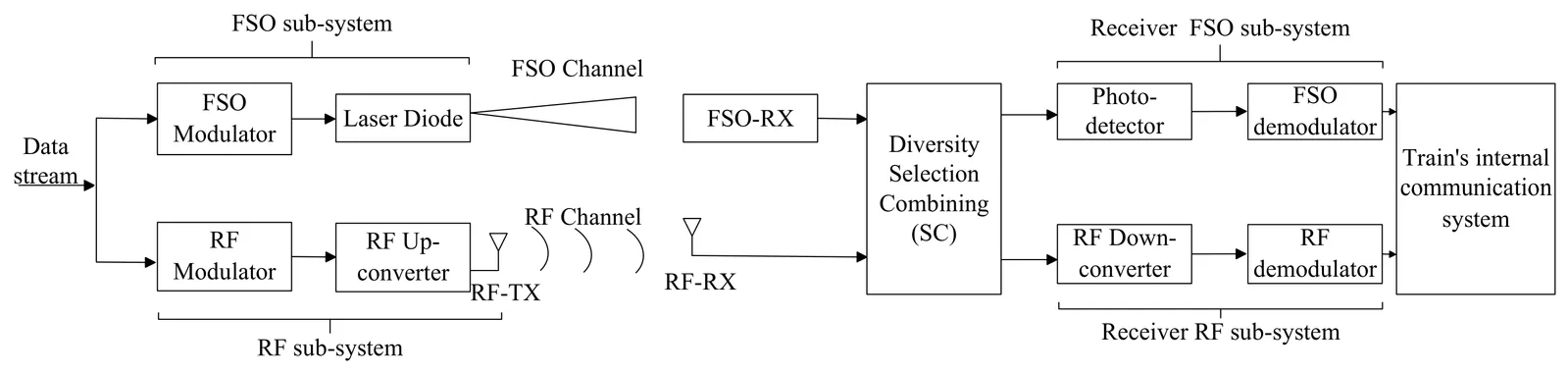
The proposed hybrid FSO/RF communication system diagram for HSR scenarios.

Figure 3 depicts the coverage configuration and the overall architecture of the proposed hybrid FSO/RF communication system in HSR scenarios. The data stream delivered through the optical backbone is first processed at the hybrid transmitter, where on-off keying (OOK) modulation is adopted for the FSO link and binary phase-shift keying (BPSK) modulation is applied to the RF link. The modulated signals are subsequently transmitted through the corresponding FSO and RF channels.

The FSO subsystem is based on intensity modulation and direct detection (IM/DD). To ensure non-negative optical intensity, a direct-current bias is introduced into the modulated waveform by the laser diode before optical transmission [23]. The biased optical signal propagates through the FSO channel and is converted into an electrical signal by the photodetector at the receiver. After bias removal and signal demodulation, the recovered information is delivered to the in-train communication system, enabling end-to-end connectivity between the base station and onboard users.

The received electrical signal at the FSO receiver is modeled as [24]

(1) y=Rhx+n

where $x\in \{0,\;2P_{\mathrm{tx}}\}$ represents the OOK-modulated optical intensity, with the two symbols 0 and $2P_{\mathrm{tx}}$ being transmitted with equal probability. Here, $P_{\mathrm{tx}}$ denotes the average optical transmit power. The parameter *R* is the photoelectric conversion efficiency, *h* is the overall FSO channel gain, and *n* represents the additive white Gaussian noise (AWGN) term. The noise component is statistically independent of the transmitted signal and follows a zero-mean Gaussian distribution with variance $\sigma_{n}^{2}$. The FSO channel gain is given by

(2) $$h=h_{l}h_{a}h_{p}$$

where $h_{l}$, $h_{a}$, and $h_{p}$ denote the atmospheric attenuation, atmospheric turbulence, and pointing error components, respectively. These three components characterize the dominant propagation impairments in HSR FSO links, including atmospheric effects and beam misalignment induced by high mobility and mechanical disturbances, and are modeled as follows.

During free-space optical transmission, the optical signal suffers from atmospheric attenuation caused by absorption and scattering. The absorption loss originates mainly from interactions between the optical wave and atmospheric molecules, whereas scattering effects are associated with particles such as molecules, aerosols, and water droplets in fog, haze, rain, and snow. Generally, atmospheric scattering can be categorized into Mie scattering and Rayleigh scattering. Since Mie scattering dominates within the typical FSO wavelength range, the influence of Rayleigh scattering is neglected in this work. By jointly considering the effects of atmospheric absorption, scattering, and adverse weather conditions, the atmospheric attenuation factor $h_{l}$ in (2) is modeled as [25]

(3) $$h_{l}=e^{-m(\lambda )l}$$

where m(λ) denotes the wavelength-dependent attenuation coefficient determined by the atmospheric visibility and optical wavelength, which is given by

(4) $$m\left(\lambda \right)=\frac{17.35}{V}{\left(\frac{\lambda }{550}\right)}^{-q}$$

where *V* (in km) is the visibility distance obtained from trackside visibility measurements, and λ represents the wavelength of the transmitted optical signal. The parameter *q* describes the size distribution characteristics of atmospheric scattering particles under different weather conditions and is expressed as [25]

(5) $$q=\left\{\begin{array}{cc} 1.6, & \mathrm{if}\;V>50\;\mathrm{km},\\ 1.3, & \mathrm{if}\;6\;\mathrm{km}<V<50\;\mathrm{km},\\ 0.16V+34, & \mathrm{if}\;1\;\mathrm{km}<V<6\;\mathrm{km},\\ V-0.5, & \mathrm{if}\;0.5\;\mathrm{km}<V<1\;\mathrm{km},\\ 0, & \mathrm{if}\;V<0.5\;\mathrm{km}. \end{array}\right.$$

Consequently, the atmospheric attenuation under various weather conditions can be evaluated using (3)–(5).

In addition to atmospheric attenuation, turbulence-induced intensity fluctuations significantly affect FSO link performance. To characterize the random variations of received optical intensity, several turbulence models have been developed. Among them, the log-normal and Gamma–Gamma models are widely adopted for weak and strong turbulence regimes, respectively. The Málaga distribution provides a unified model for different turbulence regimes and has been widely adopted for characterizing irradiance fluctuations under various atmospheric turbulence conditions in FSO systems [26]. Accordingly, the probability density function (PDF) of the atmospheric turbulence coefficient $h_{a}$ in (2) is given by

(6) $$f_{h_{a}}\left(h_{a}\right)=A_{1}\sum_{k=1}^{\beta }a_{k}h_{a}^{\frac{\alpha +k}{2}-1}K_{\alpha -k}\left(2\sqrt{\frac{\alpha \beta h_{a}}{\xi_{g}\beta +\Omega^{\prime }}}\right)$$

where the coefficients $A_{1}$ and $a_{k}$ are defined as

(7) A1≜2αα2ξg1+α2Γ(α)ξgβξgβ+Ω′β+α2,ak≜β−1k−1ξgβ+Ω′Γ(k)1−k2Ω′ξgk−1αβk2.

Here, α characterizes the effective number of large-scale scattering cells, $\xi_{g}$ represents the average power of the scattered optical component, and β is the turbulence-related fading parameter. In addition, $\Omega^{\prime }$ denotes the average power of the coherent optical component, Γ(·) is the Gamma function, and $K_{v}\left(\cdot \right)$ refers to the modified Bessel function of the second kind of order *v*.

Furthermore, pointing deviations resulting from mechanical vibrations and train-induced oscillations may cause additional power fluctuations in FSO transmissions. The PDF of the pointing error coefficient $h_{p}$ in (2) is expressed as [26]

(8) $$f_{h_{p}}\left(h_{p}\right)=\frac{r^{2}}{A_{0}^{r^{2}}}h_{p}^{r^{2}-1},\;0\leq h_{p}\leq A_{0}$$

where *r* represents the ratio between the equivalent beam radius at the receiver plane and the standard deviation of the pointing displacement. The parameter $A_{0}$ is defined as ${\left[\mathrm{erf}\left(\sqrt{\pi }B/2\sqrt{2}w_{L}\right)\right]}^{2}$, where $w_{L}=w_{0}\sqrt{1+{\left[\lambda L/\left(\pi w_{0}^{2}\right)\right]}^{2}}$ denotes the beam radius at propagation distance *L* corresponding to the $e^{-2}$ intensity level. Here, $w_{0}$ is the initial beam waist radius, and erf(·) denotes the error function. The pointing error component provides a statistical characterization of beam misalignment caused by train vibration, mechanical instability, and other mobility-related disturbances. Detailed beam acquisition and tracking procedures are not explicitly modeled, since this work focuses on statistical coverage performance analysis rather than instantaneous beam control design.

With the established FSO channel model, the instantaneous received SNR at the FSO receiver is given by

(9) $$\gamma_{1}=\frac{2P_{\mathrm{tx}}^{2}R^{2}h^{2}}{\sigma_{n}^{2}}=\frac{2P_{\mathrm{tx}}^{2}R^{2}}{\sigma_{n}^{2}}{\left(h_{l}h_{a}h_{p}\right)}^{2}$$

### 2.3. RF Subsystem

As shown in Figure 3, at the transmitter of the RF subsystem, the BPSK-modulated signal is up-converted to the RF carrier frequency of 800 MHz before being transmitted over the RF link. Considering the deterministic railway trajectory and the availability of train position and velocity information, the Doppler shift caused by the relative motion between the train and RF-BS can be predicted and compensated. Therefore, the residual Doppler effect after compensation is neglected in the RF channel model. At the RF receiver, the incoming RF waveform is converted to the baseband and demodulated to retrieve the transmitted information. The recovered data are subsequently delivered to the in-train communication system, enabling end-to-end connectivity between the base station and onboard users. The received RF signal is modeled as [21]

(10) $$y=\sqrt{E}h_{\mathrm{RF}}x_{\mathrm{RF}}+n$$

where *E* represents the transmit power, $x_{\mathrm{RF}}$ denotes the BPSK-modulated symbol with normalized average power, and $h_{\mathrm{RF}}$ is the RF channel coefficient. The noise term *n* is assumed to be AWGN with zero mean and variance $\sigma_{n}^{2}$, and is statistically independent of the transmitted signal.

To capture the propagation characteristics of HSR RF links, a composite channel model consisting of path loss, shadow fading, and small-scale fading is adopted. Accordingly, the RF channel coefficient is expressed as

(11) $$h_{\mathrm{RF}}=g\sqrt{PL(l)S}$$

where PL(l), *S*, and *g* represent the path loss gain, shadow fading factor, and small-scale fading coefficient, respectively. These components are individually modeled to describe the large-scale and small-scale propagation effects in HSR environments.

The path loss gain is given by [21]

(12) $$PL\left(l\right)=10^{-\frac{A+B\log_{10}\left(l\right)}{10}}$$

where the parameters *A* and *B* are defined as

(13) $$\left\{\begin{array}{c} A=\Delta_{1}+74.52+26.16\log_{10}\left(f\right)-13.82\log_{10}\left(h_{b}\right)-3.2{\left(\log_{10}\left(11.75h_{T}\right)\right)}^{2},\\ B=44.9-6.55\log_{10}\left(h_{b}\right)+\Delta_{2} \end{array}\right.$$

where *f* (MHz) denotes the carrier frequency, and $h_{b}$ (m) and $h_{T}$ (m) represent the effective antenna heights of the RF-BS and onboard receiver, respectively. The parameters $\Delta_{1}$ and $\Delta_{2}$ are environment-specific correction factors.

The shadow fading term *S* is modeled as a log-normal random variable, with the PDF given by

(14) $$f_{S}\left(s\right)=\frac{\xi }{\sqrt{2\pi }\sigma s}\exp\left(-\frac{{\left(10\log_{10}s\right)}^{2}}{2\sigma^{2}}\right),\;s>0$$

where ξ=10/ln(10) and σ represents the standard deviation of the shadow fading component in dB. The shadow fading component statistically captures large-scale variations caused by environmental obstructions and propagation environment changes.

The small-scale fading component *g* is modeled as Rayleigh fading, which is widely adopted for HSR communication scenarios due to the existence of multipath propagation. Since *g* follows a Rayleigh distribution, $g^{2}$ follows an exponential distribution. Assuming $E[g^{2}]=1$, the PDF of $g^{2}$ can be expressed as

(15) $$f_{g^{2}}\left(t\right)=e^{-t},\;t\geq 0$$

The combination of Rayleigh small-scale fading and log-normal shadow fading results in a Suzuki composite fading channel, also referred to as a Rayleigh–lognormal distribution. This model is widely adopted for describing RF propagation scenarios involving both multipath effects and large-scale environmental obstructions in HSR communications [27].

Accordingly, the instantaneous received SNR of the RF link is obtained as

(16) $$\gamma_{2}=\frac{Eg^{2}PL\left(l\right)S}{\sigma_{n}^{2}}$$

## 3. Coverage Performance Analysis

This section first characterizes the statistical behavior of the received SNR, followed by an evaluation of the HSR coverage performance in terms of ECP and the percentage of CCA.

### 3.1. Statistical Characteristics of SNR

#### 3.1.1. CDF of SNR for FSO Link

The PDF of *h* is given by

(17) $$f_{h}\left(h\right)=\int f_{h|h_{a}}(h\left|h_{a}\right)f_{h_{a}}\left(h_{a}\right)dh_{a}$$

where $f_{h|h_{a}}\left(h|h_{a}\right)$ is the conditional probability given the turbulence state $h_{a}$. From [26], we have

(18) $$\begin{array}{cc} f_{h}\left(h\right) & \;=\int_{h/\left(h_{l}A_{0}\right)}^{\infty }f_{h_{a}}\left(h_{a}\right)\frac{h}{h_{l}h_{a}}f_{h_{p}}\;\left(\frac{h}{h_{l}h_{a}}\right)dh_{a}\\ \; & \;=\int_{h/\left(h_{l}A_{0}\right)}^{\infty }A_{1}\sum_{k=1}^{\beta }a_{k}h_{a}^{\frac{\alpha +k}{2}-1}K_{a-k}\;\left(2\sqrt{\frac{\alpha \beta h_{a}}{\xi_{g}\beta +\Omega^{\prime }}}\right)\frac{h}{h_{l}h_{a}}\frac{r^{2}}{A_{0}^{r^{2}}}{\left(\frac{h}{h_{l}h_{a}}\right)}^{r^{2}-1}dh_{a}\\ \; & \;=\frac{r^{2}A_{1}}{2h}\sum_{k=1}^{\beta }b_{k}G_{1,3}^{3,0}\left(\frac{\alpha \beta h}{\left(\xi_{g}\beta +\Omega^{\prime }\right)A_{0}e^{-m(\lambda )l}}\left|\begin{array}{c} 1+r^{2}\\ \begin{array}{c} r^{2},\;\alpha ,\;k \end{array} \end{array}\right.\right) \end{array}$$

where $b_{k}=a_{k}{\left[\alpha \beta /(\xi_{g}\beta +\Omega^{\prime })\right]}^{-\frac{\alpha +k}{2}}$, $G_{p,q}^{m,n}\left(\cdot \right)$ denotes the Meijer G-function in the standard notation. Based on the relationship between the instantaneous SNR $\gamma_{1}$ and the channel gain *h* in (3), the CDF of $\gamma_{1}$ can be obtained via random variable transformation in the following theorem.

**Theorem** **1.** *The cumulative distribution function (CDF) of $\gamma_{1}$ can be expressed as*

(19) $$F_{\gamma_{1}}\left(\gamma_{1}\right)=\frac{r^{2}A_{1}}{2}\sum_{k=1}^{\beta }b_{k}G_{2,4}^{3,1}\left(\frac{\sigma_{n}\alpha \beta \gamma_{1}e^{m(\lambda )l}}{\left(\xi_{g}\beta +\Omega^{\prime }\right)\sqrt{2}P_{\mathrm{tx}}RA_{0}}\left|\begin{array}{c} \begin{array}{c} 1,\; \end{array}1+r^{2}\\ \begin{array}{c} r^{2},\;\alpha ,\;k,\;0 \end{array} \end{array}\right.\right)$$

**Proof.**  See Appendix A. □

#### 3.1.2. CDF of SNR for RF Link

According to [21], the PDF of $\gamma_{2}$ is

(20) $$f_{\gamma_{2}}\left(\gamma_{2}\right)=\frac{\sigma_{n}^{2}}{\sqrt{\pi }E\cdot PL\left(l\right)}\sum_{i=1}^{N_{p}}H_{i}10^{-\frac{\sqrt{2}\sigma t_{i}}{10}}\exp\left(-\frac{\sigma_{n}^{2}\gamma_{2}10^{-\frac{\sqrt{2}\sigma t_{i}}{10}}}{E\cdot PL(l)}\right)$$

where $N_{p}$ denotes the order of the Gauss–Hermite quadrature, and $t_{i}$ and $H_{i}$ represent the corresponding quadrature abscissas and weights, respectively. Combined with the ECP expression in [21], the CDF of $\gamma_{2}$ is given by

(21) $$F_{\gamma_{2}}\left(\gamma_{2}\right)=1-\frac{1}{\sqrt{\pi }}\sum_{i=1}^{N_{p}}H_{i}\exp\left(-\frac{\sigma_{n}^{2}\gamma_{2}10^{-\frac{\sqrt{2}\sigma t_{i}}{10}}}{E\cdot PL(l)}\right)$$

### 3.2. ECP Analysis

This subsection investigates the ECP performance of the hybrid FSO/RF HSR communication system under the SC scheme. The FSO and RF channels are assumed to be statistically independent since they are dominated by different channel impairments and physical mechanisms.

#### 3.2.1. Definition of ECP

The ECP represents the probability that the received SNR at the cell edge (l=D) remains above a predefined threshold. The ECP is given by

(22) $$\begin{array}{cc} P_{e}\left(\gamma \right) & \;=\mathrm{Pr}\left\{\gamma \left(D\right)>r_{\mathrm{th}}\right\}\\ \; & \;=1-\mathrm{Pr}\left\{\gamma \left(D\right)\leq r_{\mathrm{th}}\right\}\\ \; & \;=1-F_{\gamma }\left(\gamma \left(D\right)=r_{\mathrm{th}}\right) \end{array}$$

#### 3.2.2. ECP of SC-Based Hybrid FSO/RF System

Among various diversity combining techniques, SC selects the branch providing the maximum instantaneous SNR for signal detection due to its low implementation complexity. Accordingly, the output SNR of the SC receiver is given by [28]

(23) $$\gamma =\max\left(\gamma_{1},\gamma_{2}\right)$$

According to the selection criterion in (23), the distribution of the SC output SNR can be obtained by combining the statistical characteristics of the FSO and RF links. Since the two links are assumed to be statistically independent, the CDF of γ is formulated as the product of their individual CDFs. Based on this result, the ECP of the hybrid FSO/RF HSR system is further derived in closed form, as summarized in Theorem 2.

**Theorem** **2.** *The ECP of the SC-based hybrid FSO/RF HSR communication system can be expressed as follows*

(24) $$\begin{array}{cc} P_{e}\left(\gamma \right) & \;=1-F_{\gamma }\left(\gamma =r_{\mathrm{th}}\right)\\ \; & \;=1-\frac{r^{2}A}{2}\sum_{k=1}^{\beta }b_{k}G_{2,4}^{3,1}\left(\frac{\sigma_{n}\alpha \beta r_{\mathrm{th}}e^{m(\lambda )D}}{\left(\xi_{g}\beta +\Omega^{\prime }\right)\sqrt{2}P_{\mathrm{tx}}RA_{0}}\left|\begin{array}{c} 1,1+r^{2}\\ r^{2},\alpha ,k,0 \end{array}\right.\right)\\ \; & \;+\frac{r^{2}A}{2\sqrt{\pi }}\sum_{i=1}^{N_{p}}H_{i}\exp\left(-\frac{\sigma_{n}^{2}r_{\mathrm{th}}10^{-\frac{\sqrt{2}\sigma t_{i}}{10}}}{E\cdot PL(l)}\right)\sum_{k=1}^{\beta }b_{k}G_{2,4}^{3,1}\left(\frac{\sigma_{n}\alpha \beta r_{\mathrm{th}}e^{m(\lambda )D}}{\left(\xi_{g}\beta +\Omega^{\prime }\right)\sqrt{2}P_{\mathrm{tx}}RA_{0}}\left|\begin{array}{c} 1,1+r^{2}\\ r^{2},\alpha ,k,0 \end{array}\right.\right) \end{array}$$

**Proof.**  See Appendix B. □

**Corollary** **1.** *By substituting (19) into (22), the ECP of the FSO subsystem can be derived in closed form as follows*

(25) $$P_{e}\left(\gamma_{1}\right)=1-\frac{r^{2}A^{2}}{2}\sum_{k=1}^{\beta }b_{k}G_{2,4}^{3,1}\;\left(\frac{\sigma_{n}\alpha \beta r_{\mathrm{th}}e^{m(\lambda )D}}{\left(\xi_{g}\beta +\Omega^{\prime }\right)\sqrt{2}P_{\mathrm{tx}}RA_{0}}\;|\;\begin{array}{c} 1,\;1+r^{2}\\ r^{2},\;\alpha ,\;k,\;0 \end{array}\right)$$

**Remark** **1.** *The ECP of the RF subsystem has been derived in [21] and is given by*

(26) $$P_{e}\left(\gamma_{2}\right)=\frac{1}{\sqrt{\pi }}\sum_{i=1}^{N_{p}}H_{i}\exp\;\left(-\frac{\sigma_{n}^{2}r_{\mathrm{th}}10^{-\frac{\sqrt{2}\sigma_{t_{i}}}{10}}}{E\cdot PL(D)}\right)$$

### 3.3. Percentage of CCA Analysis

This subsection focuses on the analysis of the percentage of CCA, which serves as an additional metric for evaluating the spatial coverage performance of the proposed HSR communication system.

#### 3.3.1. Definition of Percentage of CCA

For narrow-strip HSR cells, the percentage of CCA characterizes the average coverage reliability along the railway trajectory and is defined as

(27) $$\begin{array}{cc} P_{a} & \;=\frac{1}{D}\int_{0}^{D}\mathrm{Pr}\left\{\gamma \left(l\right)\geq r_{\mathrm{th}}\right\}\;dl\\ & \;=\frac{1}{D}\int_{0}^{D}P_{e}\left(l\right)\;dl \end{array}$$

#### 3.3.2. Percentage of CCA for the SC-Based Hybrid FSO/RF System

Based on the definition of the percentage of CCA in (27), the percentage of CCA can be interpreted as the spatially averaged performance of the SC-based system along the train trajectory. By substituting the ECP expression of the SC scheme given in (A6) into (30), the percentage of CCA can be reformulated as an integral over the propagation distance.

However, the resulting expression involves both Meijer G function terms and nonlinear exponential attenuation terms, which makes the integral intractable to evaluate in closed form. To address this issue, a variable transformation together with a power-law approximation is introduced to obtain a tractable analytical expression.

Based on these manipulations, a closed-form expression for the percentage of CCA can be derived, as stated in Theorem 3.

**Theorem** **3.** *The percentage of CCA for the SC-based hybrid FSO/RF HSR communication system can be expressed as follows*

(28) $$\begin{array}{cc} P_{a}^{\mathrm{SC}} & \;=1-\frac{r^{2}A}{2Dm(\lambda )}\sum_{k=1}^{\beta }b_{k}[G_{3,5}^{3,2}\left(\frac{\sigma_{n}\alpha \beta r_{\mathrm{th}}e^{m(\lambda )D}}{\left(\xi_{g}\beta +\Omega^{\prime }\right)\sqrt{2}P_{\mathrm{tx}}RA_{0}}\left|\begin{array}{c} 1,\;1,\;1+r^{2}\\ r^{2},\;\alpha ,\;k,\;0,\;0 \end{array}\right.\right)\\ \; & \;\;-G_{3,5}^{3,2}\left(\frac{\sigma_{n}\alpha \beta r_{\mathrm{th}}}{\left(\xi_{g}\beta +\Omega^{\prime }\right)\sqrt{2}P_{\mathrm{tx}}RA_{0}}\left|\begin{array}{c} 1,\;1,\;1+r^{2}\\ r^{2},\;\alpha ,\;k,\;0,\;0 \end{array}\right.\right)]\\ \; & \;\;+\frac{Cr^{2}A}{2Dm\left(\lambda \right)\sqrt{\pi }}[\sum_{i=1}^{N_{p}}H_{i}{\left(e^{m(\lambda )D}\right)}^{S}\sum_{k=1}^{\beta }b_{k}G_{3,5}^{3,2}\left(\frac{\sigma_{n}\alpha \beta r_{\mathrm{th}}e^{m(\lambda )D}}{\left(\xi_{g}\beta +\Omega^{\prime }\right)\sqrt{2}P_{\mathrm{tx}}RA_{0}}\left|\begin{array}{c} 1-S,\;1,\;1+r^{2}\\ r^{2},\;\alpha ,\;k,\;0,\;-S \end{array}\right.\right)\\ \; & \;\;-\sum_{i=1}^{N_{p}}H_{i}\sum_{k=1}^{\beta }b_{k}G_{3,5}^{3,2}\left(\frac{\sigma_{n}\alpha \beta r_{\mathrm{th}}}{\left(\xi_{g}\beta +\Omega^{\prime }\right)\sqrt{2}P_{\mathrm{tx}}RA_{0}}\left|\begin{array}{c} 1-S,\;1,\;1+r^{2}\\ r^{2},\;\alpha ,\;k,\;0,\;-S \end{array}\right.\right)] \end{array}$$

**Proof.**  See Appendix C. □

**Remark** **2.** *The percentage of CCA of the FSO subsystem can be expressed as [6]*

(29) $$\begin{array}{cc} P_{a}^{\mathrm{FSO}} & \;=1-\frac{r^{2}A_{1}}{2Dm(\lambda )}\sum_{k=1}^{\beta }b_{k}[G_{3,5}^{3,2}\;\left(\frac{\sigma_{n}\alpha \beta r_{\mathrm{th}}e^{m(\lambda )D}}{\left(\xi_{g}\beta +\Omega^{\prime }\right)\sqrt{2}P_{\mathrm{tx}}RA_{0}}\left|\begin{array}{c} 1,\;1,\;1+r^{2}\\ r^{2},\;\alpha ,\;k,\;0,\;0 \end{array}\right.\right)\\ & \;\;-G_{3,5}^{3,2}\;\left(\frac{\sigma_{n}\alpha \beta r_{\mathrm{th}}}{\left(\xi_{g}\beta +\Omega^{\prime }\right)\sqrt{2}P_{\mathrm{tx}}RA_{0}}\left|\begin{array}{c} 1,\;1,\;1+r^{2}\\ r^{2},\;\alpha ,\;k,\;0,\;0 \end{array}\right.\right)] \end{array}$$

**Remark** **3.** *The percentage of CCA of the RF subsystem can be expressed as [21]*

(30) $$P_{a}^{\mathrm{RF}}=\frac{1}{\sqrt{\pi }D}\frac{10}{B}\sum_{i=1}^{N_{p}}H_{i}{\left(\frac{E}{N_{0}r_{\mathrm{th}}}10^{\frac{\sqrt{2}\sigma t_{i}-A}{10}}\right)}^{\frac{10}{B}}c\left(\frac{10}{B},\frac{N_{0}r_{\mathrm{th}}}{E}10^{\frac{A-\sqrt{2}\sigma t_{i}}{10}D^{\frac{B}{10}}}\right)$$

*where $c\left(a,b\right)=\int_{0}^{b}t^{a-1}e^{-t}dt$ denotes the lower incomplete gamma function.*

## 4. Numerical Results

This section provides numerical evaluations of the proposed hybrid FSO/RF HSR communication system to assess its cell coverage performance. Monte Carlo simulations are conducted to examine the consistency of the analytical results. The close match between the theoretical predictions and simulation outcomes demonstrates the effectiveness of the derived coverage expressions. The key simulation parameters are summarized in Table 1.

### 4.1. Results of ECP

**Figure 4:**
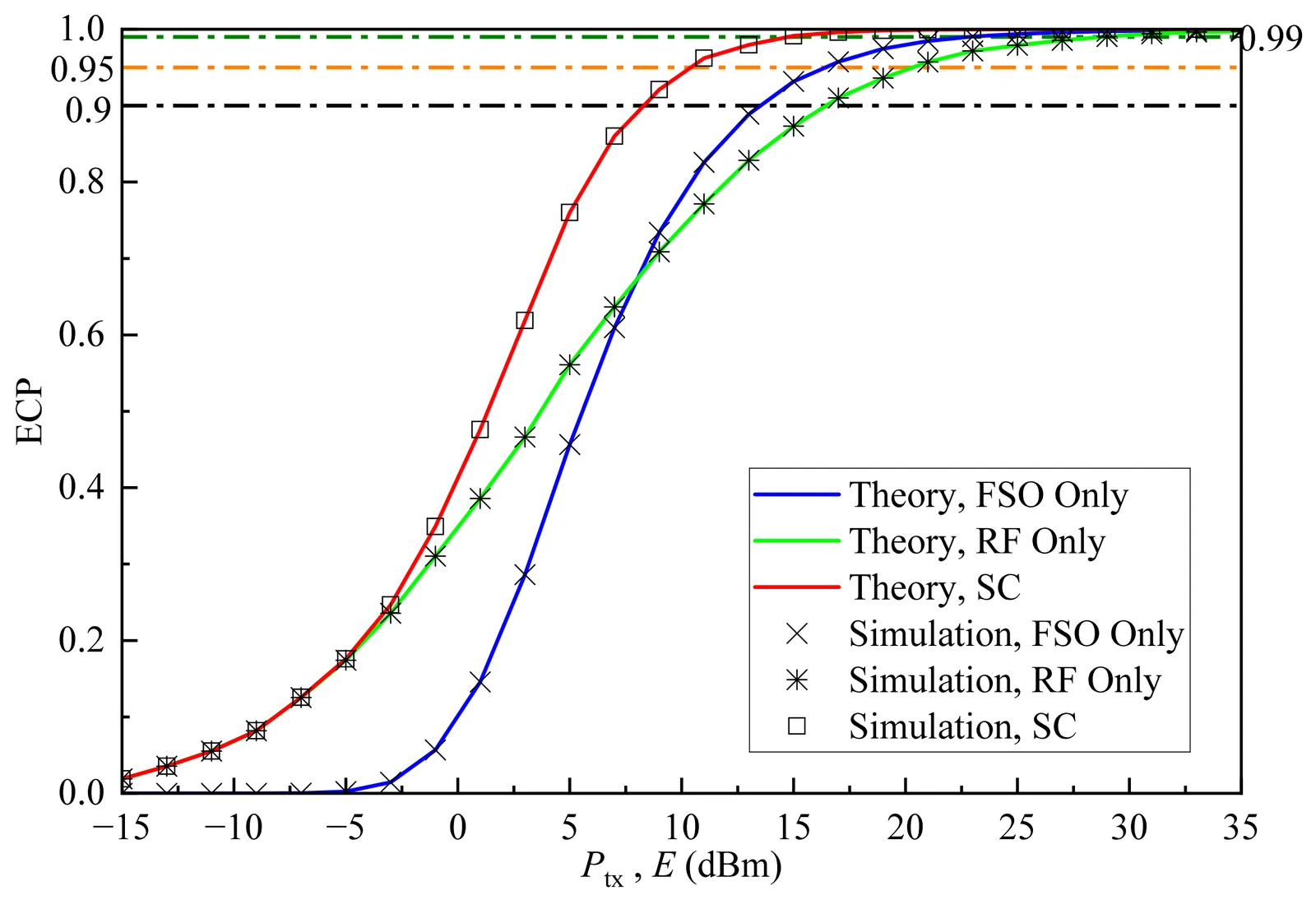
The relationship between the ECP and the transmit power when D=1 and V=30 km.

**Figure 5:**
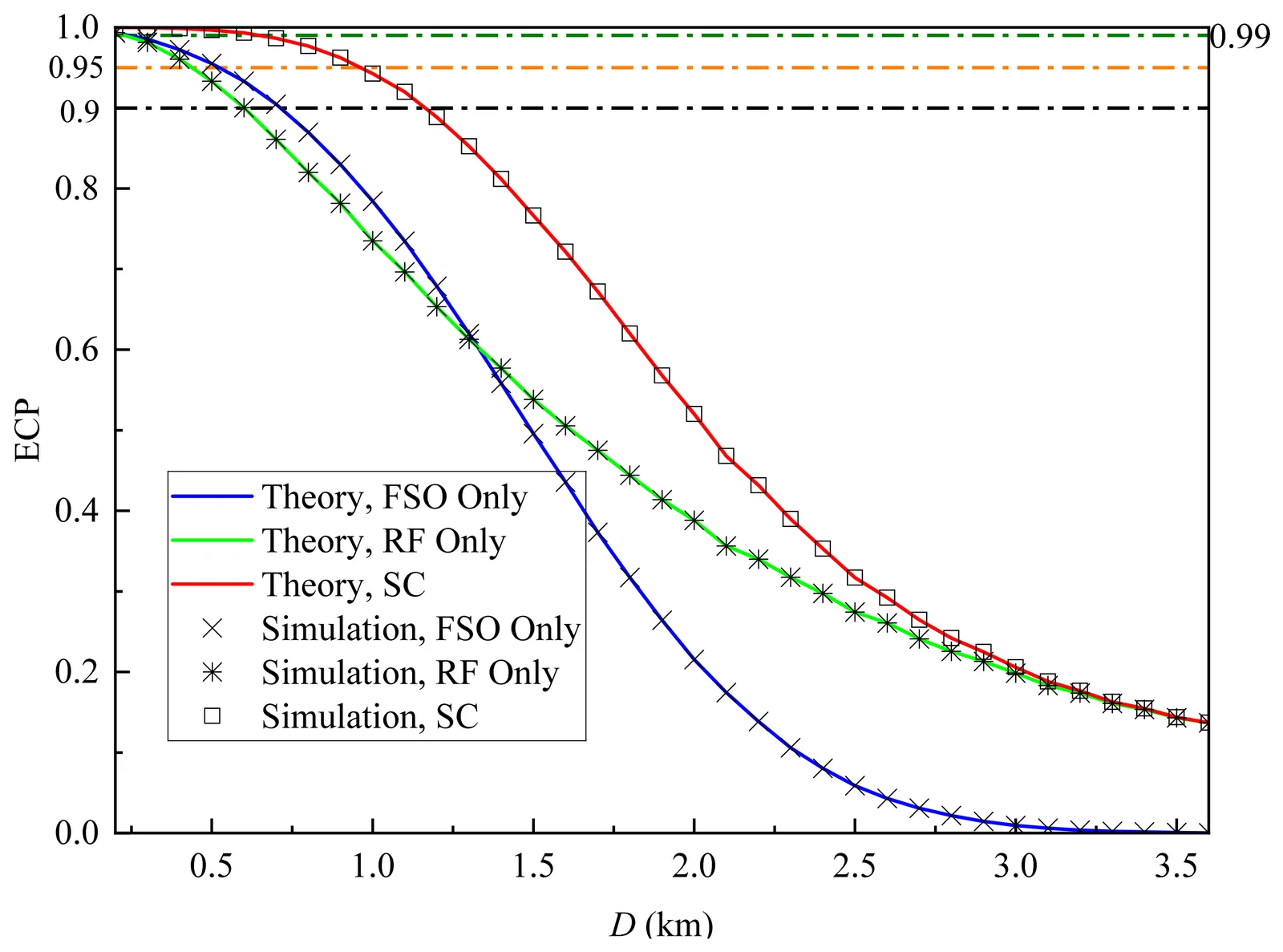
The relationship between the ECP and the cell diameter when $P_{\mathrm{tx}}=E=10\;\mathrm{dBm}$ and V=30km.

**Figure 6:**
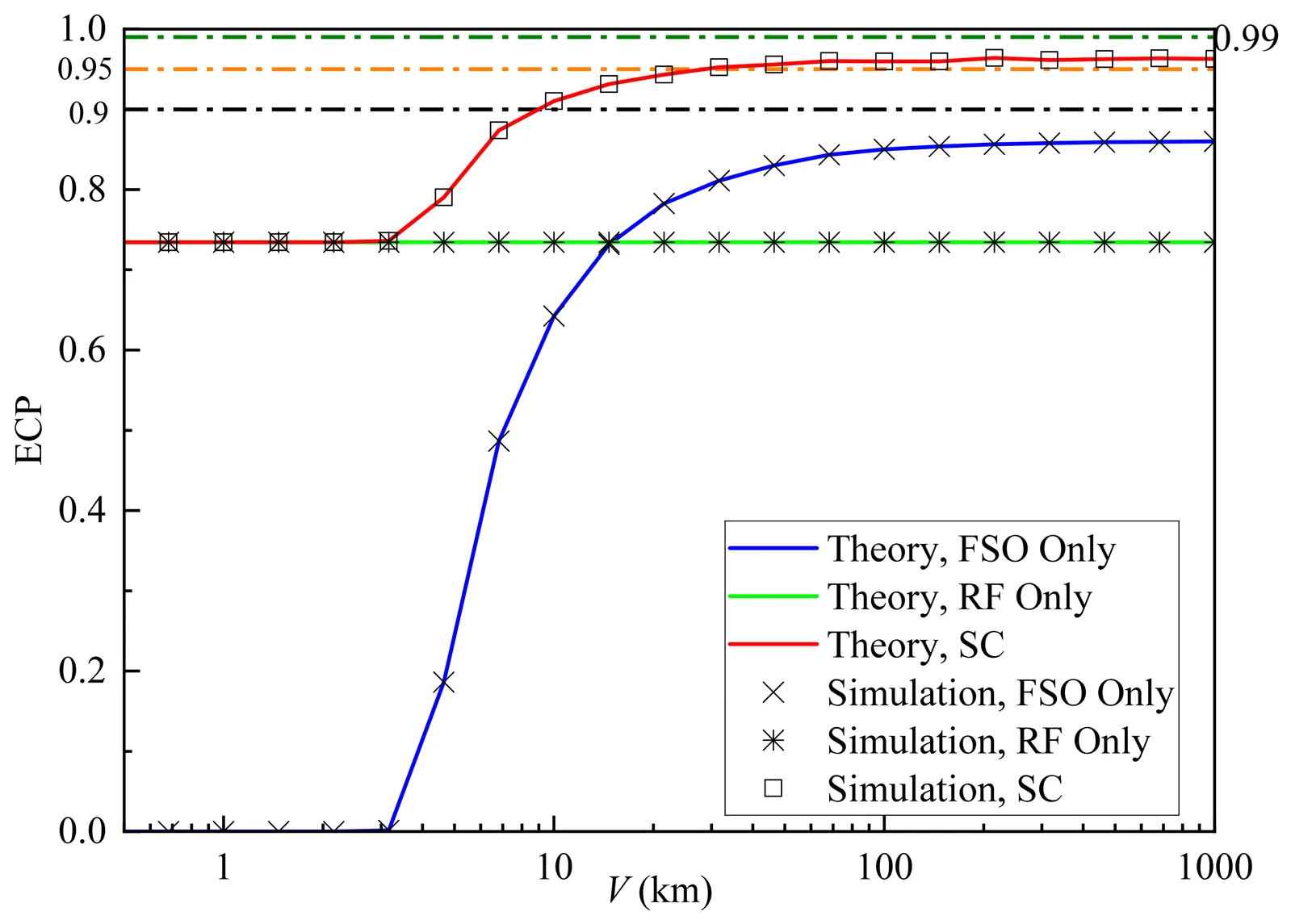
The relationship between the ECP and atmospheric visibility when $P_{\mathrm{tx}}=E=10\;\mathrm{dBm}$ and D=1km.

**Figure 7:**
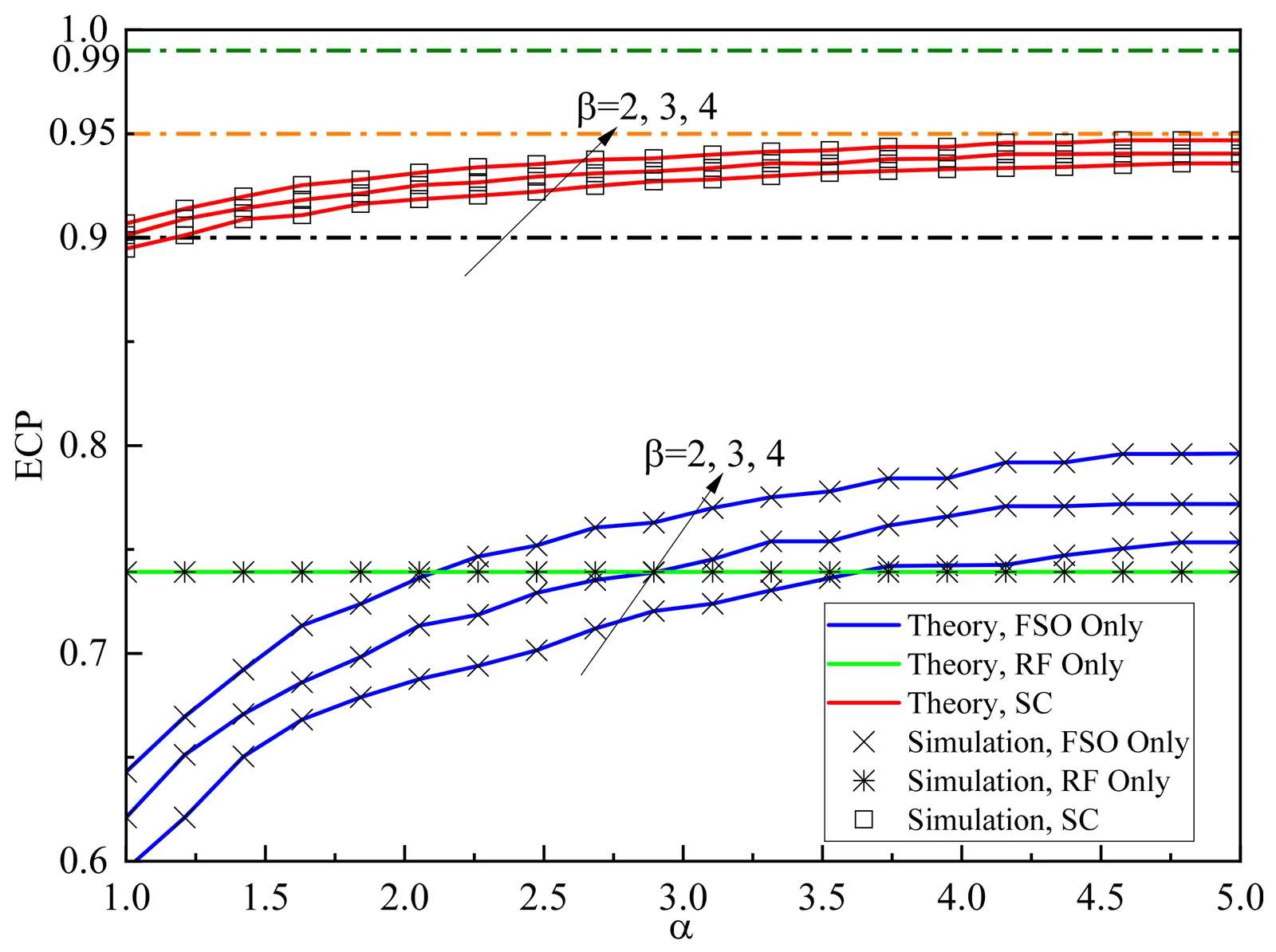
The relationship between the ECP and Málaga turbulence parameter α for different values of β when $P_{\mathrm{tx}}=E=10\;\mathrm{dBm}$, D=1 km, and V=30 km.

This subsection presents the ECP performance shown in Figure 4, Figure 5, Figure 6 and Figure 7. Three transmission schemes are evaluated for comparison, including the standalone FSO link, the standalone RF link, and the hybrid FSO/RF system employing the SC strategy. According to the parameters listed in Table 1, the FSO link operates at a wavelength of 850 nm. This near-infrared wavelength is selected due to its favorable safety characteristics and practical applicability in public environments. In addition, Class 1 or Class 1M laser sources are assumed, which comply with international laser safety requirements and are considered safe for normal human exposure [29,30].

Since the considered hybrid FSO/RF SC communication system for high-speed rail requires a high level of service reliability, we further plot the ECP curves at 90%, 95%, and 99% levels for comparison [31]. The same levels are also considered in the CCA results. It can be seen that, by appropriately selecting the transmit power, the system can satisfy different ECP requirements, indicating that the considered scheme exhibits strong stability and reliability in terms of coverage performance.

Figure 4 illustrates the ECP versus transmit power for different schemes. It can be observed that the ECP of all three schemes increases monotonically with transmit power and gradually saturates, since higher transmit power improves the received SNR and reduces the outage probability. Notably, the RF link achieves higher ECP in the low-power region, while the FSO link gradually outperforms the RF link as the transmit power increases. This is because RF links are less sensitive to atmospheric effects and provide more reliable edge coverage under power-limited conditions, whereas FSO links can exploit their high directivity and gain advantages when sufficient power is available. The SC scheme achieves the best ECP over the entire power range by dynamically selecting the link with higher instantaneous SNR, thereby obtaining diversity gain and mitigating the impact of deep fading in individual links.

Figure 5 illustrates the ECP versus cell diameter for different schemes. It can be observed that the ECP of all three schemes decreases with increasing cell diameter. This is because a larger cell diameter results in longer propagation distances, introducing higher path loss and reducing the received SNR, which increases the outage probability. The FSO link exhibits a faster ECP degradation than the RF link, since the effects of atmospheric turbulence and pointing errors become more significant as the distance increases. In contrast, the RF link benefits from stronger diffraction and scattering capabilities and shows better robustness against distance variation. The SC scheme achieves the highest ECP over the entire cell diameter range because it benefits from the higher link quality of the FSO link at short distances, while relying on the RF link to mitigate FSO degradation at longer distances, thereby reducing the impact of single-link fading on edge coverage reliability.

Figure 6 illustrates the ECP versus atmospheric visibility for different schemes. It can be observed that the ECP of the RF link remains almost unchanged with visibility variation, whereas the ECP of the FSO link increases significantly with increasing visibility and gradually saturates under high-visibility conditions. This is because low visibility (e.g., dense fog) strengthens atmospheric scattering, causing severe attenuation of the FSO optical signal, while the RF link is much less affected by visibility degradation. Moreover, the SC scheme achieves the highest ECP over the entire visibility range. Under low-visibility conditions, SC mainly relies on the RF link to maintain edge reliability; as visibility improves, SC can exploit the improved FSO link quality to further enhance the ECP. This improvement is attributed to the complementary weather-dependent characteristics of FSO and RF links, allowing SC to dynamically select the link with better channel conditions and mitigate the reliability degradation caused by adverse weather.

Figure 7 illustrates the ECP versus the Málaga turbulence parameter α for different schemes under different β values. Since the RF link is unaffected by atmospheric turbulence, its ECP remains almost unchanged with varying α. In contrast, the ECP of both the FSO link and the SC scheme gradually increases as α increases. This is because a larger α corresponds to weaker large-scale turbulence fluctuations, which reduces optical intensity scintillation and mitigates signal fading, thereby improving the received optical signal quality. Moreover, increasing β further enhances the ECP, since a larger β represents weaker small-scale scattering effects and reduces the impact of turbulence-induced fading on the FSO link. The SC scheme achieves higher ECP over the entire α range because it can compensate for FSO degradation through the RF link under strong turbulence conditions and exploit the higher received SNR of the FSO link under weak turbulence conditions, thereby further improving edge coverage reliability.

For Figure 4, Figure 5, Figure 6 and Figure 7, the simulation results are in excellent agreement with the theoretical values, with the average relative error of ECP between theoretical and simulation results being only 0.047%, thereby confirming the correctness of the derived ECP expression.

### 4.2. Results of Percentage of CCA

**Figure 8:**
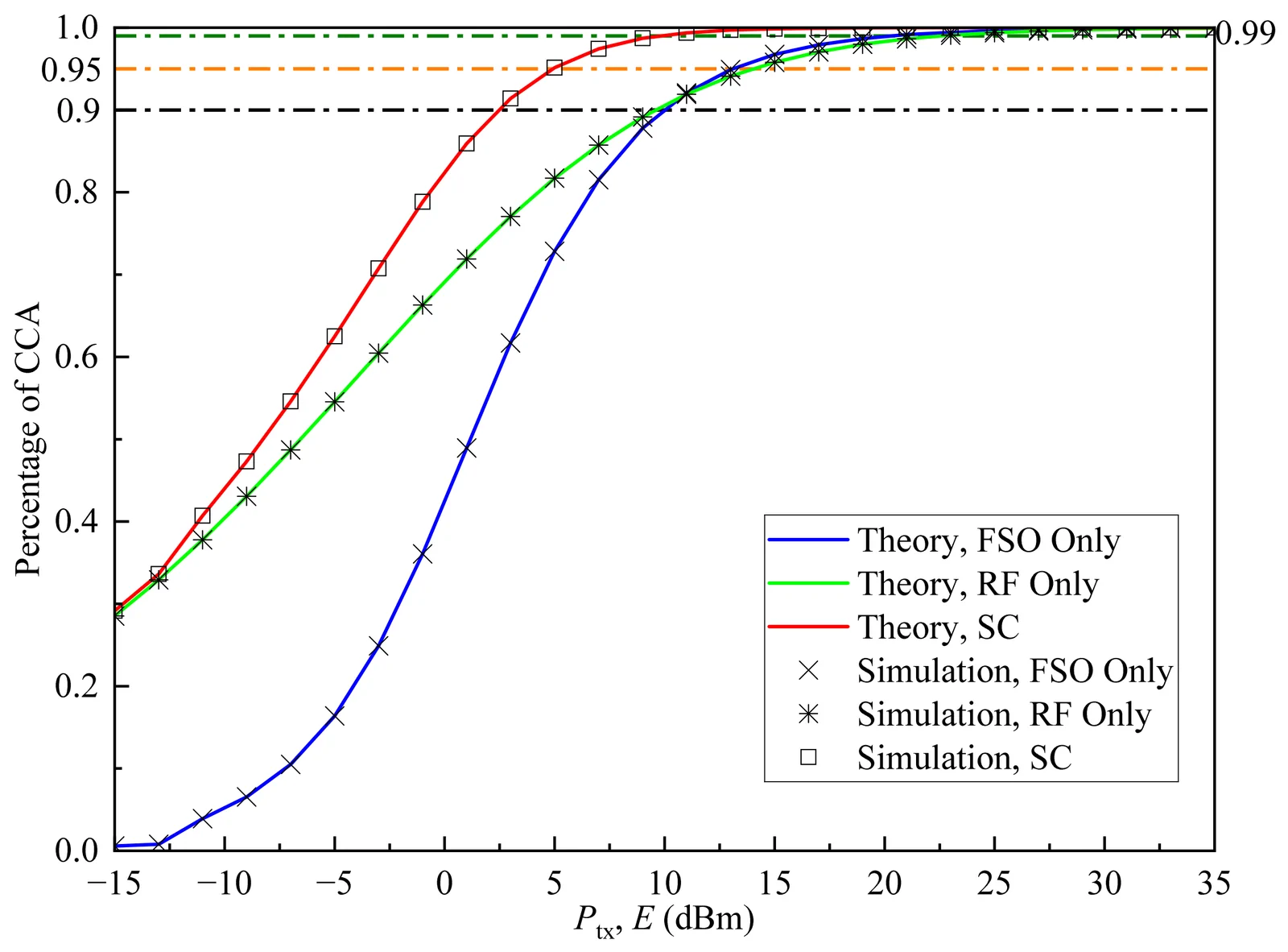
The relationship between the percentage of CCA and the transmit power when D=1 and V=30 km.

**Figure 9:**
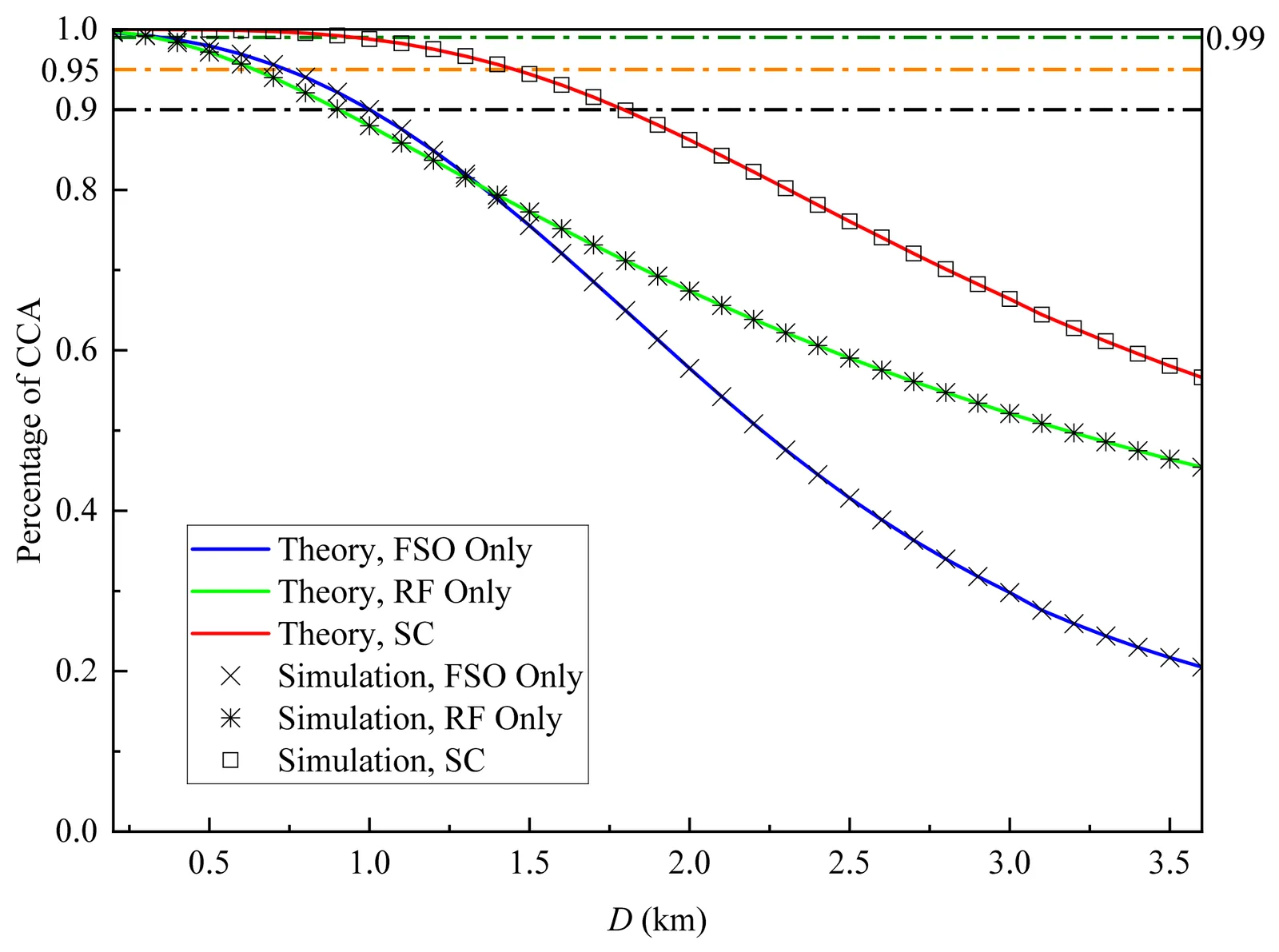
The relationship between the percentage of CCA and the cell diameter when $P_{\mathrm{tx}}=E=10\;\mathrm{dBm}$ and V=30km.

**Figure 10:**
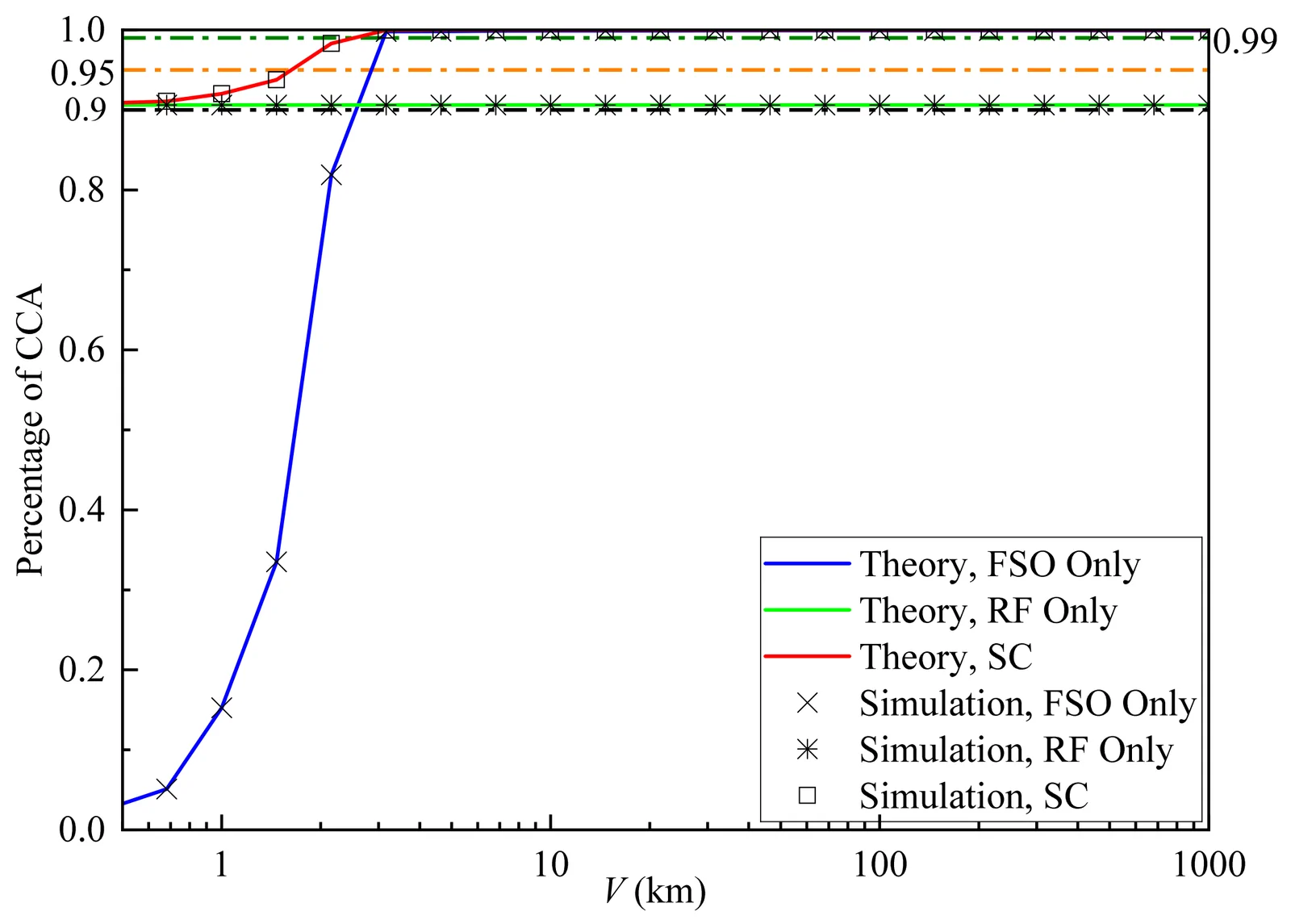
The relationship between the percentage of CCA and atmospheric visibility when $P_{\mathrm{tx}}=E=10\;\mathrm{dBm}$ and D=1km.

**Figure 11:**
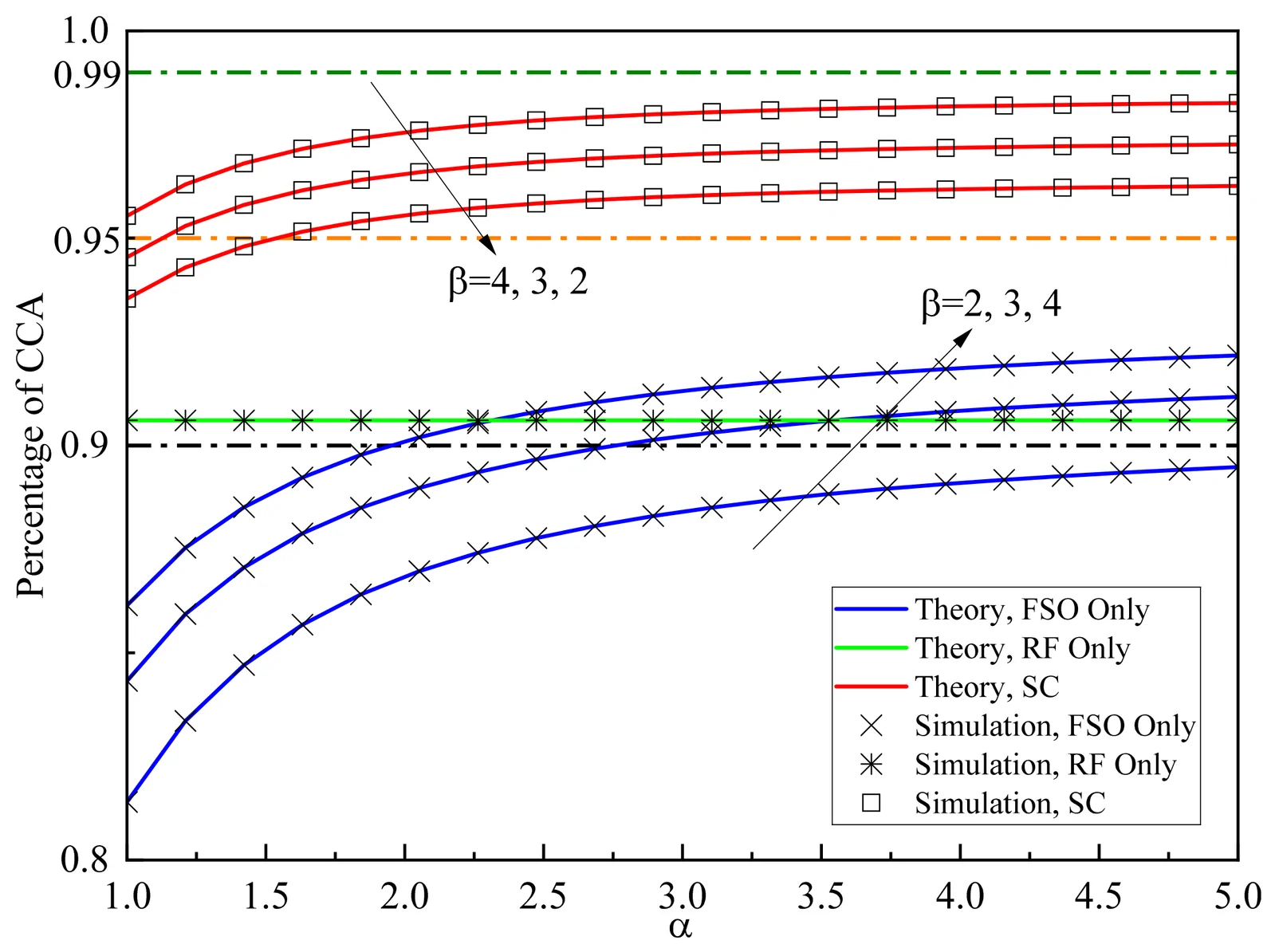
The relationship between the percentage of CCA and Málaga turbulence parameter α for different values of β when $P_{\mathrm{tx}}=E=10\;\mathrm{dBm}$, D=1 km, and V=30 km.

In this subsection, numerical results of the percentage of CCA are presented in Figure 8, Figure 9, Figure 10 and Figure 11. For comparison, the coverage performance of three schemes is considered: the FSO-only link, the RF-only link, and the hybrid FSO/RF system with the SC strategy.

Figure 8 illustrates the variation in the percentage of CCA versus transmit power for different schemes. It can be observed that the percentage of CCA for all three schemes increases monotonically with increasing transmit power and eventually saturates. This is because as the transmit power increases, more locations farther from the base station can satisfy the SNR threshold, thereby enlarging the effective coverage area. In addition, the SC scheme achieves a higher percentage of CCA over the entire power range, indicating that the hybrid link can increase the proportion of coverage area satisfying the SNR threshold. It is worth noting that the coverage expansion characteristics of the standalone FSO and RF links are different. In the low-power region, the RF link can maintain a larger coverage area satisfying the SNR threshold due to its slower propagation loss increase.

Figure 9 illustrates the variation in the percentage of CCA versus cell diameter for different schemes. It can be observed that the percentage of CCA for all three schemes decreases monotonically with increasing cell diameter. This is because an increase in cell diameter leads to longer transmission distances, causing the coverage boundary to shrink and reducing the proportion of locations satisfying the SNR threshold. Moreover, the SC scheme achieves a higher percentage of CCA over the entire distance range, especially in the medium-to-long distance region. This is because as the cell diameter increases, the longer propagation distance of the FSO link intensifies the attenuation caused by path loss, pointing errors, and atmospheric turbulence, resulting in a rapid reduction of its coverage area satisfying the SNR threshold. In contrast, the RF link is mainly affected by propagation loss and is not subject to optical attenuation, enabling more stable coverage performance over longer distances. By dynamically selecting the link with better channel conditions, SC exploits the high received SNR of the FSO link at short distances and uses the RF link to compensate for FSO degradation at longer distances, thereby increasing the percentage of CCA.

Figure 10 illustrates the variation of the percentage of CCA ratio versus atmospheric visibility for different schemes. It can be observed that the percentage of CCA for the RF link remains almost unchanged with visibility variation, whereas that of the FSO link increases significantly with increasing visibility and gradually saturates under high-visibility conditions. This is because low visibility (e.g., dense fog) enhances atmospheric scattering, resulting in severe attenuation of the FSO optical signal, while the RF link is less affected by fog-induced attenuation. Moreover, the SC scheme achieves a higher percentage of CCA over the entire visibility range. Under low-visibility conditions, SC mainly relies on the RF link to maintain a larger effective coverage area. As visibility improves, the atmospheric attenuation of the FSO link decreases, allowing SC to select the FSO link with higher received SNR and further enlarge the coverage area satisfying the SNR threshold. This improvement mainly benefits from the complementary weather-dependent characteristics of the FSO and RF links. By selecting the link with better channel conditions, SC can increase the effective coverage ratio under different visibility conditions.

Figure 11 presents the percentage of CCA versus the Málaga turbulence parameter α under different β values. It can be observed that the percentage of CCA of the FSO link and the SC scheme increases with increasing α. This is because a larger α indicates weaker large-scale turbulence, which reduces optical intensity fluctuations and enlarges the area satisfying the SNR threshold. Moreover, increasing β further improves the percentage of CCA by mitigating the impact of small-scale scattering-induced fading. The SC scheme achieves a higher CCA over the entire α range because it can utilize the RF link to compensate for the coverage loss of the FSO link under strong turbulence conditions, thereby maintaining a larger area satisfying the SNR threshold.

For Figure 8, Figure 9, Figure 10 and Figure 11, the average relative error of the percentage of CCA between theoretical and simulation results is 0.095%; therefore, the gap is small and can be ignored. This verifies the correctness of the derived percentage of CCA expression.

## 5. Conclusions

This paper investigated a hybrid FSO/RF communication system for narrow strip-like high-speed railway cells by incorporating an SC-based reception strategy and a location-aware base-station activation mechanism. The coverage performance of the proposed architecture was analyzed through theoretical derivations and Monte Carlo simulations. The main conclusions are summarized as follows:

- A hybrid FSO/RF coverage model for HSR scenarios was established by considering heterogeneous along-track base-station deployment and the major propagation impairments of FSO and RF links. Based on this model, analytical expressions for ECP and the percentage of CCA were derived, and their accuracy was verified through Monte Carlo simulations.
- The SC-based hybrid architecture consistently outperforms standalone FSO and RF schemes in terms of ECP and the percentage of CCA. This improvement is attributed to the ability of SC to dynamically select the link with better channel conditions, thereby exploiting the complementary characteristics of FSO and RF links under different transmission distances and atmospheric conditions.
- The numerical results demonstrate that system coverage is affected by transmit power, cell diameter, and atmospheric visibility. The FSO link provides higher performance under favorable propagation conditions, while the RF link offers more stable performance under adverse conditions or longer transmission distances. The proposed location-aware activation mechanism enables adaptive base-station operation based on train positions and provides potential energy-saving benefits by avoiding unnecessary activation of non-serving base stations.

The current work focuses on the theoretical coverage analysis of hybrid FSO/RF HSR systems. Future studies may further consider practical factors, such as detailed energy consumption models, imperfect positioning information, handover latency, blockage effects, and curved railway trajectories, to evaluate system performance under more realistic deployment conditions. 

## Figures and Tables

**Table 1:** Main simulation parameters.

Parameters	Symbols	Values
Photoelectric conversion efficiency	*R*	0.8
Receiver diameter	*B*	0.2 m
Divergence angle	θ	0.01 rad
Noise standard deviation	$\sigma_{n}$	$10^{-6.5}$ A/Hz
Beam wavelength	λ	850 nm
Effective number of large-scale cells	α	3.99
Amount of fading parameter	β	2
Pointing error parameter	*r*	5
Average scattered optical power	$\xi_{g}$	0.2
Average coherent optical power	$\Omega^{\prime }$	0.5
Beam waist radius	$w_{0}$	$10^{-4}$
Standard deviation of $10\log^{s}$	σ	6
Carrier frequency	*f*	800 MHZ
BS antenna height	$h_{b}$	20 m
TAP antenna height	$h_{T}$	4 m
Order of Gauss-Hermite approximation	$N_{p}$	40
Number of Snapshots	*N*	$10^{5}$

## Data Availability

The original contributions presented in this study are included in the article. Further inquiries can be directed to the corresponding author.

## Abbreviations

The following abbreviations are used in this manuscript:

5G	Fifth-generation
AWGN	Additive white Gaussian noise
BPSK	Binary phase-shift keying
BSs	Base stations
CCA	Cell coverage area
CDF	Cumulative distribution function
ECP	Edge coverage probability
FSO	Free-space optical
FSO-BSs	Free-space optical Base Stations
FSO-RX	Free-space optical Receiver
HSR	High-speed railway
OOK	On-off keying
PDF	Probability density function
RF	Radio frequency
RF-BSs	Radio frequency Base Stations
RF-RX	Radio frequency Receiver
SC	Selection combining
SNR	Signal-to-noise ratio

## Appendix A. Proof of Theorem 1

From (9) and (18), and applying the random variable transformation theorem, the PDF of $\gamma_{1}$ can be obtained as follows

(A1) $$f_{\gamma_{1}}\left(\gamma_{1}\right)=\frac{r^{2}A}{4\gamma_{1}}\sum_{k=1}^{\beta }b_{k}G_{1,3}^{3,0}\left(\frac{\alpha \beta \sigma_{n}\sqrt{\gamma_{1}}}{\left(\xi_{g}\beta +\Omega^{\prime }\right)\sqrt{2}P_{\mathrm{tx}}RA_{0}e^{-m(\lambda )l}}\left|\begin{array}{c} 1+r^{2}\\ \begin{array}{c} r^{2},\;\alpha ,\;k \end{array} \end{array}\right.\right)$$

Subsequently, the CDF of $\gamma_{1}$ is obtained as

(A2) $$\begin{array}{cc} F_{\gamma_{1}}\left(\gamma_{1}\right) & \;=\int_{-\infty }^{\gamma_{1}}f_{\gamma_{1}}\left(t\right)\;dt\\ \; & \;=\int_{-\infty }^{\gamma_{1}}\frac{r^{2}A}{4t}\sum_{k=1}^{\beta }b_{k}G_{1,3}^{3,0}\;\left(\frac{\alpha \beta \sigma_{n}\sqrt{t}}{\left(\xi_{g}\beta +\Omega^{\prime }\right)\sqrt{2P_{\mathrm{tx}}}\;RA_{0}e^{-m(\lambda )l}}\left|\begin{array}{c} 1+r^{2}\\ r^{2},\;\alpha ,\;k \end{array}\right.\right)dt \end{array}$$

By letting $t=x^{2}$ and performing a change of variables, we obtain

(A3) $$\begin{array}{c} F_{\gamma_{1}}\left(\gamma_{1}\right)=\frac{r^{2}A}{2}\int_{-\infty }^{\gamma_{1}}x^{-1}\sum_{k=1}^{\beta }b_{k}G_{1,3}^{3,0}\left(\frac{\alpha \beta \sigma_{n}x}{\left(\xi_{g}\beta +\Omega^{\prime }\right)\sqrt{2}P_{\mathrm{tx}}RA_{0}e^{-m(\lambda )l}}\left|\begin{array}{c} 1+r^{2}\\ \begin{array}{c} r^{2},\;\alpha ,\;k \end{array} \end{array}\right.\right)dx \end{array}$$

Finally, by applying ([29], eq.(07.34.21.0002.01)) the closed-form expression in (19) is obtained.

## Appendix B. Proof of Theorem 2

Assuming that the FSO and RF links are statistically independent, the CDF of the SC output SNR can be expressed as [28]

(A4) $$F_{\gamma }\left(\gamma \right)=F_{\gamma_{1}}\left(\gamma_{1}\right)F_{\gamma_{2}}\left(\gamma_{2}\right)$$

By substituting (19) and (21) into the (A4), the CDF of the SC output SNR can be obtained as follows

(A5) $$\begin{array}{cc} F_{\gamma }\left(\gamma \right) & \;=\frac{r^{2}A}{2}\sum_{k=1}^{\beta }b_{k}G_{2,4}^{3,1}\left(\frac{\sigma_{n}\alpha \beta \gamma_{1}e^{m(\lambda )l}}{\left(\xi_{g}\beta +\Omega^{\prime }\right)\sqrt{2}P_{\mathrm{tx}}RA_{0}}\left|\begin{array}{c} 1,1+r^{2}\\ r^{2},\alpha ,k,0 \end{array}\right.\right)\\ \; & \;\;-\frac{r^{2}A}{2\sqrt{\pi }}\sum_{i=1}^{N_{p}}H_{i}\exp\left(-\frac{\sigma_{n}^{2}r_{\mathrm{th}}10^{-\frac{\sqrt{2}\sigma t_{i}}{10}}}{E\cdot PL(l)}\right)\\ \; & \;\;\times \sum_{k=1}^{\beta }b_{k}G_{2,4}^{3,1}\left(\frac{\sigma_{n}\alpha \beta r_{\mathrm{th}}e^{m(\lambda )D}}{\left(\xi_{g}\beta +\Omega^{\prime }\right)\sqrt{2}P_{\mathrm{tx}}RA_{0}}\left|\begin{array}{c} 1,1+r^{2}\\ r^{2},\alpha ,k,0 \end{array}\right.\right) \end{array}$$

Finally, the ECP can be expressed as

(A6) $$\begin{array}{cc} P_{e}\left(\gamma \right)= & \;1-F_{\gamma }\left(\gamma =r_{\mathrm{th}}\right)\\ = & \;1-\frac{r^{2}A}{2}\sum_{k=1}^{\beta }b_{k}G_{2,4}^{3,1}\left(\frac{\sigma_{n}\alpha \beta r_{\mathrm{th}}e^{m(\lambda )D}}{\left(\xi_{g}\beta +\Omega^{\prime }\right)\sqrt{2}P_{\mathrm{tx}}RA_{0}}\left|\begin{array}{c} 1,1+r^{2}\\ r^{2},\alpha ,k,0 \end{array}\right.\right)\\ & \;+\frac{r^{2}A}{2\sqrt{\pi }}\sum_{i=1}^{N_{p}}H_{i}\exp\left(-\frac{\sigma_{n}^{2}r_{\mathrm{th}}10^{-\frac{\sqrt{2}\sigma t_{i}}{10}}}{E\cdot PL(l)}\right)\\ & \;\times \sum_{k=1}^{\beta }b_{k}G_{2,4}^{3,1}\left(\frac{\sigma_{n}\alpha \beta r_{\mathrm{th}}e^{m(\lambda )D}}{\left(\xi_{g}\beta +\Omega^{\prime }\right)\sqrt{2}P_{\mathrm{tx}}RA_{0}}\left|\begin{array}{c} 1,1+r^{2}\\ r^{2},\alpha ,k,0 \end{array}\right.\right) \end{array}$$

## Appendix C. Proof of Theorem 3

By substituting (A6) into (27), we obtain

(A7) $$\begin{array}{cc} P_{a}^{\mathrm{SC}} & \;=\frac{1}{D}\int_{0}^{D}\left[1-\frac{r^{2}A}{2}\sum_{k=1}^{\beta }b_{k}G_{2,4}^{3,1}\;\left(\frac{\sigma_{n}\alpha \beta r_{\mathrm{th}}e^{m(\lambda )l}}{\left(\xi_{g}\beta +\Omega^{\prime }\right)\sqrt{2}P_{\mathrm{tx}}RA_{0}}\left|\begin{array}{c} 1,1+r^{2}\\ r^{2},\alpha ,k,0 \end{array}\right.\right)\right]dl\\ \; & \;\;+\frac{1}{D}\int_{0}^{D}\frac{r^{2}A}{2\sqrt{\pi }}\sum_{i=1}^{N_{p}}H_{i}\exp\;\left(-\frac{\sigma_{n}^{2}r_{\mathrm{th}}10^{-\frac{\sqrt{2}\sigma t_{i}}{10}}}{E\;10^{-\frac{A+B\log_{10}\left(l\right)}{10}}}\right)\\ \; & \;\;\times \sum_{k=1}^{\beta }b_{k}G_{2,4}^{3,1}\;\left(\frac{\sigma_{n}\alpha \beta r_{\mathrm{th}}e^{m(\lambda )l}}{\left(\xi_{g}\beta +\Omega^{\prime }\right)\sqrt{2}P_{\mathrm{tx}}RA_{0}}\left|\begin{array}{c} 1,1+r^{2}\\ r^{2},\alpha ,k,0 \end{array}\right.\right)dl. \end{array}$$

After a straightforward transformation, we further have

(A8) $$\begin{array}{cc} P_{a}^{\mathrm{SC}} & \;=1-\frac{r^{2}A}{2}\sum_{k=1}^{\beta }b_{k}\int_{0}^{D}G_{2,4}^{3,1}\left(\frac{\sigma_{n}\alpha \beta r_{\mathrm{th}}e^{m(\lambda )l}}{\left(\xi_{g}\beta +\Omega^{\prime }\right)\sqrt{2}P_{\mathrm{tx}}RA_{0}}\left|\begin{array}{c} 1,1+r^{2}\\ r^{2},\alpha ,k,0 \end{array}\right.\right)dl\\ \; & \;\;+\frac{r^{2}A}{2D\sqrt{\pi }}\int_{0}^{D}\sum_{i=1}^{N_{p}}H_{i}\exp\left(-\frac{\sigma_{n}^{2}r_{\mathrm{th}}10^{-\frac{\sqrt{2}\sigma t_{i}}{10}}}{E10^{-\frac{A}{10}}10^{\log_{10}{\left(l\right)}^{-\frac{B}{10}}}}\right)\\ \; & \;\;\times \sum_{k=1}^{\beta }b_{k}G_{2,4}^{3,1}\left(\frac{\sigma_{n}\alpha \beta r_{\mathrm{th}}e^{m(\lambda )l}}{\left(\xi_{g}\beta +\Omega^{\prime }\right)\sqrt{2}P_{\mathrm{tx}}RA_{0}}\left|\begin{array}{c} 1,1+r^{2}\\ r^{2},\alpha ,k,0 \end{array}\right.\right)dl \end{array}$$

The formula can be appropriately transformed and rearranged, and further simplified to obtain

(A9) $$\begin{array}{cc} P_{a}^{\mathrm{SC}} & \;=1-\frac{r^{2}A}{2}\sum_{k=1}^{\beta }b_{k}\int_{0}^{D}G_{2,4}^{3,1}\left(\frac{\sigma_{n}\alpha \beta r_{\mathrm{th}}e^{m(\lambda )l}}{\left(\xi_{g}\beta +\Omega^{\prime }\right)\sqrt{2}P_{\mathrm{tx}}RA_{0}}\left|\begin{array}{c} 1,1+r^{2}\\ r^{2},\alpha ,k,0 \end{array}\right.\right)dl\\ \; & \;\;+\frac{r^{2}A}{2D\sqrt{\pi }}\int_{0}^{D}\sum_{i=1}^{N_{p}}H_{i}\exp\left(-\frac{\sigma_{n}^{2}r_{\mathrm{th}}10^{-\frac{\sqrt{2}\sigma t_{i}}{10}}{\left(l\right)}^{\frac{B}{10}}}{E10^{-\frac{A}{10}}}\right)\\ \; & \;\;\times \sum_{k=1}^{\beta }b_{k}G_{2,4}^{3,1}\left(\frac{\sigma_{n}\alpha \beta r_{\mathrm{th}}e^{m(\lambda )l}}{\left(\xi_{g}\beta +\Omega^{\prime }\right)\sqrt{2}P_{\mathrm{tx}}RA_{0}}\left|\begin{array}{c} 1,1+r^{2}\\ r^{2},\alpha ,k,0 \end{array}\right.\right)dl \end{array}$$

Let $t=e^{m(\lambda )l}$, then $l=\frac{\ln^{t}}{m(\lambda )}$, $dl=\frac{dt}{t\;m(\lambda )}$. Accordingly, we have

(A10) $$\begin{array}{cc} P_{a}^{\mathrm{SC}} & \;=1-\frac{r^{2}A}{2Dm(\lambda )}\sum_{k=1}^{\beta }b_{k}\int_{1}^{e^{m(\lambda )D}}t^{-1}G_{2,4}^{3,1}\left(\frac{\sigma_{n}\alpha \beta r_{\mathrm{th}}t}{\left(\xi_{g}\beta +\Omega^{\prime }\right)\sqrt{2}P_{\mathrm{tx}}RA_{0}}\left|\begin{array}{c} 1,1+r^{2}\\ r^{2},\alpha ,k,0 \end{array}\right.\right)dt\\ \; & \;\;+\frac{r^{2}A}{2Dm(\lambda )\pi }\int_{1}^{e^{m(\lambda )D}}\frac{1}{t}\sum_{i=1}^{N_{p}}H_{i}\exp\left(-\frac{\sigma_{n}^{2}r_{\mathrm{th}}{\left(\ln t\right)}^{\frac{B}{10}}}{E10^{-\frac{A}{10}}m{\left(\lambda \right)}^{\frac{B}{10}}}\right)\\ \; & \;\;\times \sum_{k=1}^{\beta }b_{k}G_{2,4}^{3,1}\left(\frac{\sigma_{n}\alpha \beta r_{\mathrm{th}}t}{\left(\xi_{g}\beta +\Omega^{\prime }\right)\sqrt{2}P_{\mathrm{tx}}RA_{0}}\left|\begin{array}{c} 1,1+r^{2}\\ r^{2},\alpha ,k,0 \end{array}\right.\right)dt \end{array}$$

To simplify the complicated analytical expression and complete the integration, the exponential term containing the logarithmic function is approximated by a simple power-law form.

Let $U=-\frac{\sigma_{n}^{2}r_{\mathrm{th}}10^{-\frac{\sqrt{2}\sigma t_{i}}{10}}}{E10^{-\frac{A}{10}}m{\left(\lambda \right)}^{\frac{B}{10}}}$, $F=e^{m(\lambda )D}$. Next, using the least-squares fitting method, the target function is given by

(A11) $$f\left(t\right)=\exp\left(U{\left(\ln^{t}\right)}^{\frac{B}{10}}\right)$$

with the fitting function written as

(A12) $$\tilde{f}\left(t\right)=Ct^{S}$$

where t∈[1,F], *U*, $\frac{B}{10}$, and *F* are constants. Our objective is to determine the optimal values of *C* and *S*.

Taking the natural logarithm on both sides yields

(A13) $$\begin{array}{c} \ln\left(f(t)\right)=U{\left(\ln^{t}\right)}^{\frac{B}{10}}\\ \ln\left(\tilde{f}\left(t\right)\right)=\ln\left(C\right)+S\ln\left(t\right) \end{array}$$

Let the variable substitution be $x=\ln\left(t\right)$, $y\left(x\right)=Ux^{\frac{B}{10}}$, $\tilde{y}\left(x\right)=\ln\left(C\right)+Sx$.

Since t∈[1,F], corresponds to the range of $\left[0,x_{\max}\right]$, where $x_{\max}=\ln\left(F\right)$, the problem is then transformed into fitting the nonlinear function $y\left(x\right)$ with a linear function $\tilde{y}\left(x\right)$ over the interval $\left[0,x_{\max}\right]$.

Define the squared-error integral as

(A14) $$E\left(S,\ln\left(C\right)\right)=\int_{0}^{x_{\max}}{\left[Ux^{\frac{B}{10}}-\left(Sx+\ln\left(C\right)\right)\right]}^{2}dx$$

To minimize the error, we take partial derivatives with respect to *S* and $\ln\left(C\right)$, and set them to zero.

Taking the partial derivative with respect to $\ln\left(C\right)$,

(A15) $$\frac{\partial E}{\partial \left(\ln\left(C\right)\right)}=-2\int_{0}^{x_{\max}}\left(Ux^{\frac{B}{10}}-Sx-\ln\left(C\right)\right)dx=0$$

Integrating yields

(A16) $$\frac{Ux_{\max}^{\frac{B}{10}+1}}{\frac{B}{10}+1}-\frac{Sx_{\max}^{2}}{2}-\left(\ln\left(C\right)\right)x_{\max}=0$$

which can be simplified as

(A17) $$\ln\left(C\right)=\frac{Ux_{\max}^{\frac{B}{10}}}{\frac{B}{10}+1}-\frac{Sx_{\max}}{2}$$

Taking the partial derivative with respect to *S*,

(A18) $$\frac{\partial E}{\partial S}=-2\int_{0}^{x_{\max}}x\left(Ux^{\frac{B}{10}}-Sx-\ln\left(C\right)\right)dx=0$$

Integrating gives

(A19) $$\frac{Ux_{\max}^{\frac{B}{10}+2}}{\frac{B}{10}+2}-\frac{Sx_{\max}^{3}}{3}-\frac{x_{\max}^{2}\ln\left(C\right)}{2}=0$$

Substituting Equation (1) into Equation (2) removes the dependence on *C*, yielding

(A20) $$\;\frac{Ux_{\max}^{B+2}}{\frac{B}{10}+2}-\frac{Sx_{\max}^{3}}{3}-\left(\frac{Ux_{\max}^{\frac{B}{10}}}{\frac{B}{10}+1}-\frac{Sx_{\max}}{2}\right)\frac{x_{\max}^{2}}{2}=0$$

After collecting like terms and simplifying, the expression for *S* is finally obtained as

(A21) $$S=\frac{6U\frac{B}{10}\cdot {\left(\ln\left(F\right)\right)}^{\frac{B}{10}-1}}{\left(\frac{B}{10}+1\right)\left(\frac{B}{10}+2\right)}$$

Substituting the obtained value of *S* into Equation (1), the corresponding expression of *C* can be derived as

(A22) $$C=\exp\left(\frac{2U\left(1-\frac{B}{10}\right){\left(\ln\left(F\right)\right)}^{\frac{B}{10}}}{\left(\frac{B}{10}+1\right)\left(\frac{B}{10}+2\right)}\right)$$

Then, by substituting *S* and *C* into (A22), we obtain

(A23) $$\begin{array}{cc} P_{a}^{\mathrm{FSO}} & \;=1-\frac{r^{2}A}{2Dm(\lambda )}\sum_{k=1}^{\beta }b_{k}\int_{1}^{e^{m(\lambda )D}}t^{-1}G_{2,4}^{3,1}\left(\frac{\sigma_{n}\alpha \beta r_{\mathrm{th}}t}{\left(\xi_{g}\beta +\Omega^{\prime }\right)\sqrt{2}P_{\mathrm{tx}}RA_{0}}\left|\begin{array}{c} 1,1+r^{2}\\ r^{2},\alpha ,k,0 \end{array}\right.\right)dt\\ \; & \;\;+\frac{Cr^{2}A}{2Dm\left(\lambda \right)\sqrt{\pi }}\int_{1}^{e^{m(\lambda )D}}\sum_{i=1}^{N_{p}}H_{i}{\left(t\right)}^{S-1}\\ \; & \;\;\times \sum_{k=1}^{\beta }b_{k}G_{2,4}^{3,1}\left(\frac{\sigma_{n}\alpha \beta r_{\mathrm{th}}t}{\left(\xi_{g}\beta +\Omega^{\prime }\right)\sqrt{2}P_{\mathrm{tx}}RA_{0}}\left|\begin{array}{c} 1,1+r^{2}\\ r^{2},\alpha ,k,0 \end{array}\right.\right)dt \end{array}$$

At this point, using ([29], eq.(07.34.21.0002.01)), we have (28).
